# How to activate threat perceptions in behavior research: A simple technique for inducing health and resource scarcity threats

**DOI:** 10.3758/s13428-024-02481-6

**Published:** 2024-08-14

**Authors:** Ozan Isler, Onurcan Yilmaz, A. John Maule, Simon Gächter

**Affiliations:** 1 School of Economics, University of Queensland, St Lucia, 4072 Australia; 2 Department of Psychology, Kadir Has University, Istanbul, 34083 Turkey; 3 Leeds University Business School, University of Leeds, Leeds, LS2 9JT UK; 4 School of Economics, University of Nottingham, Nottingham, NG7 2RD UK; 5 CESifo Munich, 81679 Munich, Germany; 6 IZA Bonn, 53113 Bonn, Germany

**Keywords:** Threat perception, Experimental manipulation technique, Health threat, Resource scarcity threat

## Abstract

Understanding our cognitive and behavioral reactions to large-scale collective problems involving health and resource scarcity threats, such as the COVID-19 pandemic, helps us be better prepared for future collective threats. However, existing studies on these threats tend to be restricted to correlational data, partly due to a lack of reliable experimental techniques for manipulating threat perceptions. In four preregistered experiments (*N* = 5152), we developed and validated an experimental technique that can separately activate perceptions of personal health threat or resource scarcity threat, either in the specific context of the COVID-19 pandemic or in general. We compared the threat manipulations to a relaxation manipulation designed to deactivate background threat perceptions as well as to a passive control condition. Confirmatory tests showed substantial activation of personal health and resource scarcity threat perceptions. This brief technique can be easily used in online experiments. Distress due to the threat manipulation was rarely reported and easily managed with a debriefing toolkit.

**Supplementary Information:**

The online version contains supplementary material available at 10.3758/s13428-024-02481-6.

## Introduction

The COVID-19 pandemic posed significant threats to both health and livelihoods. How do humans react to such existential threats? While health and resource scarcity threats can clearly affect us, often by heightening our perceptions of and emotional reactions to these threats, little is known about the systematic effects of these changes on human psychology and behavior. Most studies in behavioral and psychological sciences on the effects of health and scarcity threats rely on surveys and other correlational field data (e.g., Hensel et al., 2022; Van Bavel et al., 2022), due to a lack of experimental techniques for reliably increasing the cognitive saliency of threat perceptions. The present study addresses this methodological gap by providing a brief technique that can systematically activate perceptions of personal health or resource scarcity threats in online and laboratory studies, both in the context of the COVID-19 pandemic and in a more general threat context.  

Previous research on human threat response tended to focus on the threat of *violence* rather than health and resource scarcity threats. This work has identified a broad range of responses to violence threats including conservative reactions following terror attacks (Jost et al., 2017; Sibley et al., 2012); group divisions along ideological lines and in general (Greenberg et al., 1990; Greenberg et al., 1992; Greenberg et al., 1994; Greenberg et al., 2001), strengthening group loyalties (e.g., Van de Vyver et al., 2016); system justification motives (e.g., Ullrich & Cohrs, 2007; Van der Toorn et al., 2015); religiosity and patriotism (e.g., Bonanno & Jost 2006); authoritarianism (e.g., Echebarria-Echabe & Fernández-Guede, 2006); and support for military spending, racism, and conservatism (e.g., Craig & Richeson, 2014; Landau et al., 2004; Janoff-Bulman & Usoof-Thowfeek, 2009; Nail et al., 2009). Although these findings may be relevant for other threat types, such as scarcity and health threats, they are often based on methods predating the open science movement, which limits their generalizability due to non-experimental methods, small sample sizes, and lack of preregistration.

Environmental (e.g., scarcity), existential (e.g., mortality salience), and relational (e.g., separation) threats can affect social beliefs and outcomes (e.g., Greenberg et al., 1994; Navarrete & Fessler, 2006; Mikulincer et al., 2002; Roux et al., 2015; Tybur et al., 2014), but these findings suffer from similar limitations such as small sample sizes and unclear experimental manipulations. For instance, tasks involving viewing disgusting images or recalling memories to measure disgust sensitivity have been used to activate threat perceptions (Navarrete & Fessler, 2006; Schaller et al., 2010; Wu & Chang, 2012). However, to our knowledge, no study has systematically explored the impact of the presence (vs. absence) of photographic images, used incentivized tasks, or compared different types of threats in preregistered, high-powered experiment. In one notable exception, van Leeuwen et al. (2023) applied various techniques designed to manipulate pathogen avoidance to assess their impact on conformity, but found no significant overall effect. The null result of this comprehensive study underscores the need for effective methods to manipulate these threat perceptions in novel ways.

Reactions to existential *health* threats, compared to the threat of violence, may work through additional evolved psychological and physiological mechanisms. The behavioral immune system (BIS) is thought to have evolved to detect and respond to pathogens in the environment. Accordingly, threat detection activates the BIS, eliciting spontaneous emotional reactions that help avoid disease and prevent transmission (Ackerman et al., 2018). For example, the sight and smell of spoiled food causes disgust, motivating avoidance of potential pathogens (Terrizzi et al., 2013). This reaction enhances survival, particularly in regions with high pathogen prevalence. However, the BIS can also fuel undesirable social behaviors such as xenophobia (Helzer & Pizarro, 2011; Inbar et al., 2009; Jones & Fitness, 2008; Murray & Schaller, 2012; Wu & Chang, 2012; Faulkner et al., 2004; Fincher & Thornhill, 2012; Navarette & Fessler, 2006; Terrizzi et al., 2013). Geographical differences in pathogen prevalence are associated with more ethnocentric, collectivist, and conservative social attitudes (Murray et al., 2013; Terrizzi et al., 2013; Thornhill et al., 2009; but see Horita & Takezawa, 2018). From this perspective, the COVID-19 threat can be expected to have resulted in a global conservative shift, but to our knowledge this possibility has not been experimentally tested with manipulations reliably activating COVID-19-related threats (cf. Karwowski et al., 2020b).

The COVID-19 pandemic not only posed a serious public health threat but, through its negative impact on production processes, logistics, and financial markets, also a serious collective resource scarcity threat. Cognitive saliency of resource scarcity can influence decisions by altering perceptions of value and impairing cognitive performance (Mani et al., 2013; Mullainathan & Shafir, 2013; Shah et al., 2012; Spiller, 2011). Despite its financial implications, experiments on resource scarcity perceptions are rare, even beyond the COVID-19 context. While some studies have found effects on behavior in economic games, such as increased selfishness (Roux et al., 2015), these findings have been hard to replicate (O’Donnell et al., 2021), likely due to the reliance on small-sample studies with weak manipulation techniques (see Isler et al., 2023).

Few studies have attempted to experimentally manipulate perceived COVID-19 threat, but without distinguishing between resource scarcity and health threat perceptions. Cappelen et al. (2021) found that reminding US residents of the COVID-19 threat increased the tendency to prioritize societal problems over personal ones. This large-scale preregistered experiment aimed to activate COVID-19 threat perceptions by asking participants two straightforward questions about the pandemic: (1) “To what extent has your local community been affected by the current coronavirus crisis?” and (2) “How long do you expect the current coronavirus crisis to last?” The experimental condition with these two questions was compared to a control group that did not recive these questions. Two other experiments using a similar approach (Karwowski et al., 2020a; Karwowski et al., 2020b) involved participants reading three brief press reports. Two of the reports were on a neutral topic and were common across the conditions, whereas the third report was related to COVID-19 for the experimental group and to climate change for the control group. These reminders of COVID-19 increased anxiety without changing ideological attitudes (Karwowski et al., 2020b) or cognitive performance (Karwowski et al., 2020a). Despite these insights, these studies lack clarity regarding the construct validity of their threat manipulation techniques. In particular, it is not known to what extent health threat versus resource scarcity threat perceptions were activated and whether these contextual changes in threat type involved personal or public threat perceptions. Such systematic tests of the role of decision context in human threat response require modification of various aspects of this context (e.g., the type of threat), which our technique provides.

Current threat manipulation techniques have several limitations. First, common techniques for experimentally activating threat perceptions are either weak or ineffective. High-powered preregistered tests of some of the most commonly used techniques in psychology such as the scarcity lottery task (Krosch & Amodio, 2014), the scarcity scale task (Nelson & Morrison, 2005), and the scarcity consequences task (Roux et al., 2015) failed to successfully manipulate resource scarcity perceptions in an ongoing project (Isler et al., 2024).

Second, while the COVID-19 pandemic likely influenced both health and resource scarcity threat perceptions, this distinction is often ignored. Since these perceptions can affect behavior differently, a more accurate picture of the effects of COVID-19 can only be drawn if these two threat types are separately manipulated. It would be an added benefit if the same technique is used to construct these different manipulations, allowing comparability of the observed effects. Testing the technique’s applicability beyond the COVID-19 context could reveal whether responses are specific to COVID-19 or generalize to other contexts.

Third, existing studies often overlook the importance of background threat levels. High background threat levels during the pandemic may have resulted in control participants experiencing threat levels comparable to those subjected to threat manipulations, compromising the efficacy of experimental tests.

Fourth, and perhaps most importantly, most of the evidence on human threat response is limited to observational, correlational data, which precludes direct causal inference.

Our new technique addresses these limitations. First, recognizing that baseline threat perceptions were already high during the COVID-19 pandemic, we developed both threat manipulations and a relaxation manipulation to reduce pre-existing threat levels.

Second, we aimed to design manipulations with strong (but momentary and psychologically safe) effects on risk perceptions, addressing the weak manipulations and null results found in the literature.

Third, we distinguished between various threat types (e.g., health vs. resource scarcity, personal vs. public, COVID-19-specific vs. general) in both devising and testing our cognitive manipulation technique. This enabled systematic comparisons of responses to different threat contexts and tested the applicability of our technique.

Fourth, we tested whether the effectiveness of our technique varies with individual differences in pre-existing risk perceptions. In a field experiment, messages emphasizing self-benefit tended to increase influenza vaccination among high-risk patients, but only when they also perceived themselves at high risk (Isler et al., 2020). Since personal health threat perceptions can motivate vaccination, those who avoid the COVID-19 vaccine are likely to be less risk-averse than those who receive it.

Finally, we incorporated various pecuniary and visual design features to improve the effectiveness of our technique. Economic incentives have been shown to enhance attention to and compliance with experimental tasks (Camerer & Hogarth, 1999; Hertwig & Ortmann, 2001; Isler et al., 2023), and better compliance with task instructions can potentially increase manipulation effectiveness.

We conducted four preregistered experiments (*N* = 5152) using consistent participant selection criteria across experiments (see Section “Method”). Participants could take part in only one experiment. The first two experiments were preliminary, testing the effects of the health threat and relaxation manipulations on threat perceptions, affect, and cognitive performance. Experiment 1 tested the technique for the first time and compared various incentive schemes for increasing task compliance. Experiment 2 tested the impact of visuals on manipulation effectiveness. The final two experiments validated the effectiveness of the technique with larger samples. Experiment 3 compared the health threat and the relaxation manipulations with a control condition that measured baseline threat levels, used comprehensive outcome measures that distinguished between personal and public threat, and explored vaccination status. Experiment 4 validated the results of Experiment 3, introduced an effective COVID-19 resource scarcity threat manipulation, and tested the general applicability of these techniques beyond the COVID-19 context.

The preregistrations, datasets, analysis codes, and manipulation protocol are available at the Open Science Framework (OSF) project site, and the experimental materials are available in the Supplementary Information. The techniques developed allow for causal tests of previous correlational findings on the psychological and behavioral impact of health and scarcity threats. University of Nottingham and Kadir Has University provided ethics approvals. Informed consent was obtained from all participants.

## Experiment 1

### Method

Experiment 1 compared the COVID-19 health threat manipulation to the relaxation manipulation across three types of task incentivization using experimental procedures supported by Qualtrics (https://www.qualtrics.com/). Equal numbers of male and female participants were recruited from Prolific (https://prolific.co/), and participation was restricted to UK residents who were 18 years or older, had English as their first language, and had Prolific approval rates of 90% or above. The experiment concluded with a demographic questionnaire and a debriefing that offered support in case participants experienced distress due to the experimental manipulations. The experiment was preregistered at the OSF (https://osf.io/vgz9c).

#### Materials and procedures

**Fig. 1:**
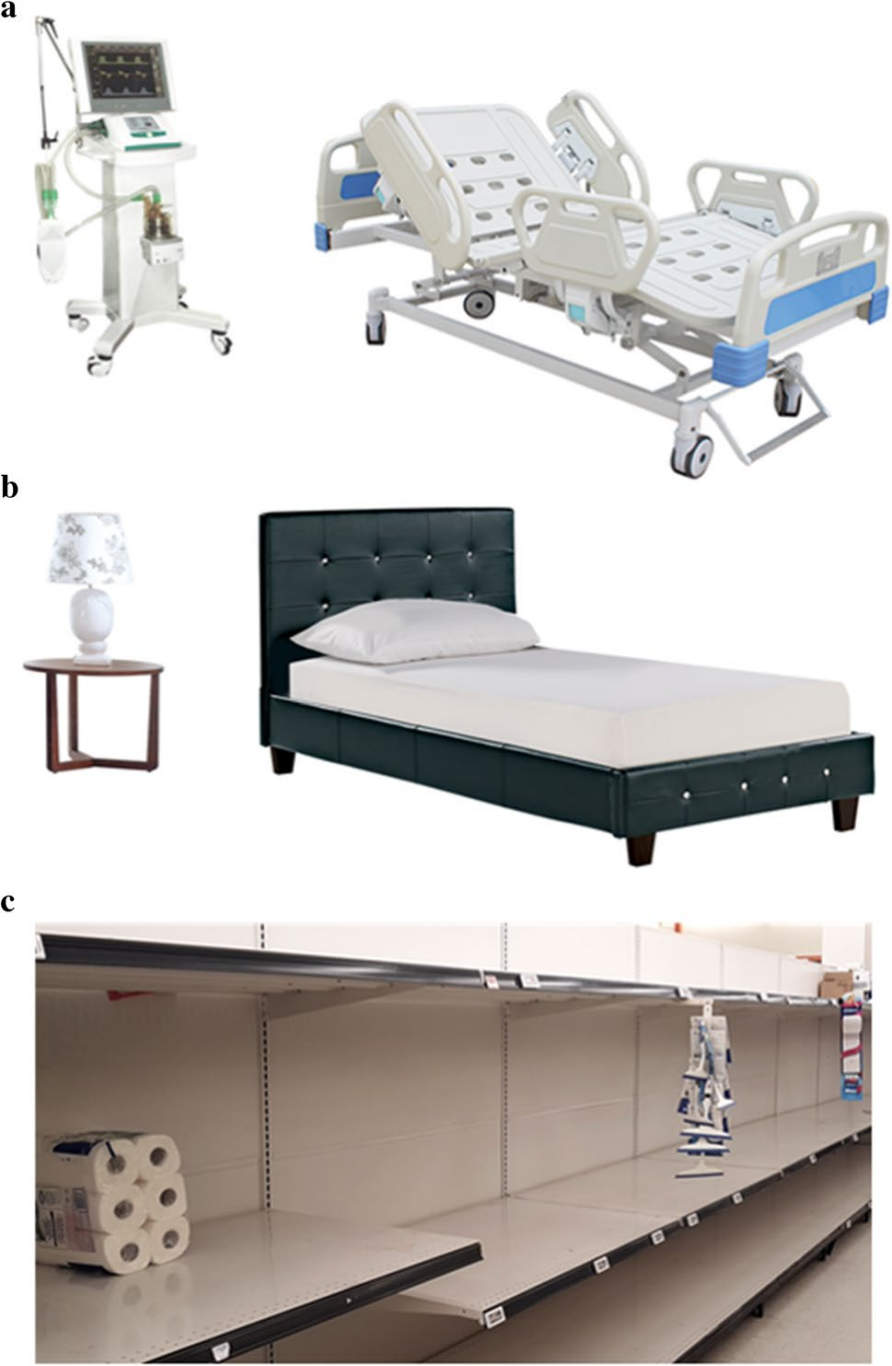
Pictures used in **a** the health threat manipulation, **b** the relaxation manipulation, and **c** the scarcity threat manipulation conditions. The picture in **c** is used in Experiment 4 (see Section "Sample"). Source: *Imgbin.com* and *Commons.wikimedia.org*. Note: Changes to written instructions were also made to distinguish between threat types

Experiment 1 used a 2 (cognitive manipulation: relaxation vs. COVID-19 health threat) by 3 (incentive type: no-bonus vs. individual bonus vs. lottery bonus) between-subjects design. To activate perceptions of health threat associated with COVID-19, a picture of an emergency hospital bedroom was displayed (see Fig. 1a) together with a sentence prompting participants to “look at the picture and think about getting very unwell from COVID-19 and needing emergency help.” A writing task was employed to activate thoughts of being severely ill due to the coronavirus. Specifically, ten seconds after the appearance of the picture and the prompt, four text boxes appeared below the picture asking participants to describe what could happen to them and how they would feel in this situation, by typing four full sentences (one in each of four separate boxes). Responses to this manipulation were compared with the relaxation manipulation, which displayed a picture of a typical single bed (see Fig. 1b). Participants were asked to think and write about lying on their bed at the end of the day and feeling very relaxed, by typing a full sentence in each of the four text boxes indicating what could happen to them and how they would feel. Participants had to enter text into all four boxes before they could continue with the study. We used a relaxation manipulation rather than a passive control condition to prevent any ceiling effects due to the severity of the ongoing COVID-19 pandemic that could have heightened baseline levels of risk perceptions. Median response times in the writing task were 143.7 seconds (s) for the relaxation manipulation condition and 161.9 s for the threat manipulation condition.

Instructions regarding incentives were provided to participants at the start of the study. Bonus incentive conditions tested whether the effects of the manipulations could be increased by motivating compliance with and attention to task instructions using individual or lottery payments. All participants received a flat fee of £0.50. In addition, participants were instructed that the individual bonus scheme paid £0.50 to everyone who had written four relevant full sentences and the lottery bonus scheme paid £5.00 to one in every ten participants if this randomly selected person had written four relevant full sentences.

Most participants complied with task instructions, as almost all text boxes (97.6%) contained three or more words. This measure did not differ statistically between the incentive conditions (Pearson’s chi-square test: *χ*^*2*^(2, *n* = 253) = 0.57, *p* = 0.752). However, bonus incentives significantly increased engagement with the task, as the average number of words written in each box was higher in the two bonus conditions than in the control (*M*_Individual-Bonus_ = 12.64 [*SD* = 5.64] vs. *M*_Control_ = 10.50 [*SD* = 4.41]: *t*(156) = 2.62, *p* = .010, *d* = 0.42; *M*_Lottery-Bonus_ = 14.94 [*SD* = 9.19] vs. *M*_Control_ = 10.50 [*SD* = 4.41]: *t*(166) = 3.80, *p* < .001, *d* = 0.59).

Threats can increase negative emotions including anger (Brooks et al., 2020) and disgust (Curtis et al., 2011; Oaten et al., 2009; Terrizzi et al., 2013). Hence, we elicited various self-reported affect measures after the COVID-19 threat manipulation (for a similar design see also Varma et al., 2020). Using a scale ranging from 1 (“very slightly or not at all”) to 5 (“extremely”), participants completed the 20-item Positive and Negative Affect Schedule (PANAS) (Watson et al., 1988). As standard, the total score on items describing positive (negative) affect constituted the *positive* (*negative*) *affect score*. On the same screen, ratings on two additional affect items (“disgusted” and “repulsed”) were elicited. The combined average of these two items was multiplied by 10 to achieve *the disgust sensitivity score*, which ranged from 0 to 50 like the positive and negative affect scores (Fincher et al., 2008; Schaller, 2011; Shook et al., 2019). Using the positive affect, the negative affect, or the disgust sensitivity scores as the dependent variable, we estimated three 2 (COVID-19 health threat) by 3 (incentive type) ANOVA models as exploratory analyses.

Next, on a scale from 0% to 100%, participants completed, in two randomly presented screens, two questions about perceived infection risk (“How likely do you think it is that, within three months from today, you [the average person in your country] will get infected by the Coronavirus (COVID-19)?”) and two questions about severity of illness (“If infected by the Coronavirus (COVID-19), how severely do you think you [the average person in your country] would have the illness?”). As preregistered, the combined average scores on these four questions constituted the perceived *threat score*, our key outcome variable that we use as a manipulation check.

To explore whether the COVID-19 threat affected cognitive performance, participants completed a multiple-choice version of the first item in the Cognitive Reflection Test (CRT, Frederick, 2005; Sirota & Juanchich, 2018), which was modified to make it less familiar to participants by referring to “a pencil and an eraser” rather than “a bat and ball.” It has been argued that people faced with mortality threats exert cognitive effort to avoid thoughts of death, potentially leading to cognitive resource scarcity and poorer performance on tasks such as the CRT (Trémolière et al., 2012, 2014). The evidence for this argument remains limited to non-preregistered studies with small sample sizes. Our sample allows for an exploration of this argument in the context of COVID-19-based mortality threat.

On the next screen, two exploratory self-reported items about reliance on intuition (“While looking at the picture and completing the four sentences...to what extent did you rely on your gut instinct?”) and about perceptions of material and financial scarcity (“…to what extent did scarcity of material or financial resources come to your mind?”) were rated on a scale from 0 (“not at all”) to 5 (“very much”). The first was similar to the CRT and explored the possible impact of threat on cognitive processing, whereas the latter was a preliminary exploration of scarcity threat to be investigated more fully in later experiments.

As in all subsequent experiments, participants in Experiment 1 completed a comprehensive debriefing, which began with an open-ended question asking whether thinking about contracting COVID-19 was distressing. All participants were provided with emergency contact lines for professional counseling as well as a Wellness Sheet that provided concrete directions for calming oneself in the event of excessive distress reactions. In Experiment 1, only 6.4% of participants in the health threat manipulation condition (7 participants) reported experiencing any distress during the experiment.

#### Sample

Using G*Power 3.1.9.4 (Faul et al., 2009), we estimated our required sample size to detect at least a medium-sized main effect of the manipulations (*f* = 0.25) in a two-way ANOVA model (1− *β* = 0.95 and *α* = 0.05) to be 251 participants in total. Experiment 1 was conducted on November 23, 2020. A total of 253 participants (age: *M* = 36.9, *SD* = 13.8) were recruited (relaxation: no-bonus = 41, individual bonus = 55, lottery bonus = 48; COVID-19 health threat: no-bonus = 32, individual bonus = 30, lottery bonus = 47).

#### Hypotheses

- H_1_: The threat scores will be higher in the health threat than in the relaxation manipulation.
- H_2_: The difference in threat scores between the health threat and the relaxation manipulations will be higher in the bonus conditions than in the no-bonus condition.

### Results

#### Confirmatory tests

No confirmatory evidence was found for either hypothesis. The preregistered two-way ANOVA model showed (H_1_) no main effect of the cognitive manipulations on the threat scores (*F*(1, 247) = 0.23, *p* = .635, η_p_^2^ < .001) and (H_2_) no interaction between these manipulations and the incentive conditions (*F*(2, 247) = 1.46, *p* = .235, η_p_^2^ = .012).

Mean threat scores in the health threat (*M*_HT_) and the relaxation manipulation (*M*_R_) conditions did not differ across the no-bonus (*M*_HT_ = 40.34, *M*_*R*_ = 40.85; *t*(71) = −0.12, *p* = .905, *d* = 0.03) and the individual bonus conditions (*M*_HT_ = 38.08, *M*_*R*_ = 40.45; *t*(83) = −0.61, *p* = .541, *d* = 0.14).^1^ In the lottery bonus condition, although the estimated effect size seems non-negligible (*d* = 0.37), this difference in threat scores between the health threat and the relaxation manipulation conditions failed to reach statistical significance at the 5% level in a two-tailed *t*-test (*M*_HT_ = 36.96, *M*_*R*_ = 30.96; *t*(93) = 1.80, *p* = .075).

#### Exploratory analysis

We explored the effect of the COVID-19 health threat manipulation on scarcity perceptions, affect, and cognitive performance here and in the subsequent experiments, because the perception of threat can influence emotions and decision-making (e.g., Trémolière et al., 2012, 2014).

##### Threat perceptions

Although perceptions of resource scarcity were on average higher in the health threat than in the relaxation manipulation (*M*_HT_ = 3.13, *M*_*R*_ = 2.40), this difference also failed to reach statistical significance at the 5% level in a two-tailed *t*-test (*t*(251) = 1.91, *p* = .058, *d* = 0.24).

##### Affect

Compared to the relaxation manipulation, the health threat manipulation increased negative affect (*M*_HT_ = 24.27, *M*_*R*_ = 11.85; *t*(251) = 15.40, *p* < .001, *d* = 1.95), decreased positive affect (*M*_HT_ = 19.43, *M*_*R*_ = 22.35; *t*(251) = −3.02, *p* = .003, *d* = 0.38), and increased disgust sensitivity (*M*_HT_ = 16.06, *M*_*R*_ = 10.90; *t*(251) = 6.42, *p* < .001, *d* = 0.81). These affect measures did not vary significantly between the incentive conditions (one-way ANOVAs: *p*s ≥ .255).

##### Cognitive performance

No significant effect of the threat manipulations was found on either the single CRT item (*M*_HT_ = 0.37, *M*_*R*_ = 0.30; *t*(251) = 1.15, *p* = .253, *d* = 0.15) or the self-reported reliance on intuition (*M*_HT_ = 7.05, *M*_*R*_ = 6.78; *t*(251) = 0.83, *p* = .409, *d* = 0.10).

### Discussion

Experiment 1 was a preliminary study designed to detect medium or larger effects. Individual and lottery bonus incentives increased task engagement, but they did not substantially improve manipulation effectiveness. While the promise of the lottery bonus incentive schemes should be investigated further, in the following experiments we continue to use individual bonus incentive schemes for their simplicity. The cognitive manipulations changed affect as expected but, except for the lottery bonus condition, failed to have a discernible impact on threat perceptions. We surmised that these failures could stem from three design features: (1) eliciting the affect measures first, with 22 items in total, could have diluted the effect of the cognitive manipulations on threat perception measures; (2) asking about “the average person in one’s country” to measure threat perceptions could have resulted in objective risk estimates rather than measures of spontaneous threat perceptions; and/or (3) showing a picture of the hospital bed in the health threat manipulation might have limited the effectiveness of the manipulation by constraining natural thought processes, thereby diluting negative thoughts. Hence, in a second preliminary experiment we revised our manipulation check accordingly, placing it immediately after the threat manipulation, and additionally tested whether the presence or absence of pictures of beds (as in Fig. 1) makes a difference.

## Experiment 2

### Method

Experiment 2, preregistered at the OSF (https://osf.io/au6vj), compared the COVID-19 health threat manipulation to the relaxation manipulation condition with or without the use of pictures.

#### Materials and procedures

Experiment 2 used a 2 (cognitive manipulation: relaxation vs. COVID-19 health threat) by 2 (task type: no-picture vs. picture) between-subjects design. In the picture conditions, the cognitive manipulations were the same as Experiment 1 (Fig. 1a and b). The no-picture conditions were identical except that no pictures were displayed and the instructions were modified by removing any reference to pictures. Median response times in the writing task were 143.5 s for the relaxation manipulation and 164.9 s for the health threat manipulation condition. Most text boxes (98.6%) contained three or more words, with an average of 13.60 words per text box.

Next, participants completed a modified manipulation check involving a question on health threat perceptions and a question on scarcity threat perceptions on a scale ranging from 0 to 100: “While making an assessment and trying to construct sentences…” (1) “...to what extent did risks to your personal health come to your mind?” and (2) “...to what extent did scarcity of material resources (such as lack of goods and services) or scarcity of financial resources (such as inadequate income or savings) come to your mind?” The average scores across these two questions about personal health and resource scarcity threat perceptions constituted the perceived *threat score* that we use in confirmatory tests. PANAS and disgust sensitivity items used in Experiment 1 were elicited next, followed in the second part of the study by the same CRT item used in Experiment 1.

Participants were paid a flat fee of £0.50. We used random lottery incentives, a standard protocol for determining individual bonus payments in experimental economic research (Starmer & Sugden, 1991). Accordingly, the participants were told that there were two parts to the study, one of which would be selected to determine their additional earnings. If the first part was chosen (i.e., the cognitive manipulation and the following manipulation check), then participants earned an additional £0.50 for writing four full relevant sentences (i.e., the same as the individual bonus condition of Experiment 1). If the second part was chosen, then participants earned an additional £0.50 for correctly answering the CRT item. In the debriefing, 12.8% of participants in the threat manipulation condition (28 participants) reported experiencing distress during the experiment.

#### Sample

Based on exploratory evidence in Experiment 1, we estimated our required sample size using G*Power 3.1.9.4 (Faul et al., 2009) to detect at least a small-to-medium main effect of the cognitive manipulations (*f* = 0.175) in a two-way ANOVA model (1− *β* = 0.95 and *α* = 0.05) to be 427 participants in total. Experiment 2 was conducted on November 27, 2020. A total of 433 participants (age: *M* = 37.1, *SD* = 14.2) were recruited (relaxation: picture = 105, no-picture = 110; COVID-19 health threat: picture = 110, no-picture = 108).

#### Hypotheses

- H_3_: The threat scores will be higher in the health threat than in the relaxation manipulation.
- H_4_: The difference in threat scores between the health threat and the relaxation manipulations will depend on the task type (i.e., picture vs. no-picture).

### Results

#### Confirmatory tests

The cognitive manipulations substantially affected threat perceptions (H_3_) but there was no evidence that the pictures enhanced this effect (H_4_). The preregistered two-way ANOVA model indicated a main effect of the cognitive manipulations on the threat score (H_3_: *F*(1, 429) = 454.37, *p* < .001, η_p_^2^ = .514) with the threat scores in the health threat manipulation (*M* = 54.26) being higher than in the relaxation manipulation (*M* = 16.73) (*t*(431) = 21.32, *p* < .001, *d* = 2.05). There was no interaction between the cognitive manipulations and the task type (H_4_: *F*(1, 429) = 1.49, *p* = .223, η_p_^2^ = .003).

#### Exploratory analysis

##### Threat perceptions

The effect of the cognitive manipulations was significant for both the personal health threat (*M*_HT_ = 81.89, *M*_*R*_ = 16.05; *t*(431) = 32.20, *p* < .001) and the scarcity threat (*M*_HT_ = 26.63, *M*_*R*_ = 17.41; *t*(431) = 3.70, *p* < .001) items comprising the threat score, but the effect size for the personal health threat item (*d* = 3.09) was substantially larger than that for the scarcity threat item (*d* = 0.36).

##### Affect

Positive affect was lower (*M*_HT_ = 22.19, *M*_*R*_ = 25.55; *t*(431) = −4.50, *p* < .001, *d* = 0.43) whereas negative affect (*M*_HT_ = 23.95, *M*_*R*_ = 12.48; *t*(431) = 16.91, *p* < .001, *d* = 1.63) and disgust sensitivity (*M*_HT_ = 14.36, *M*_*R*_ = 10.98; *t*(431) = 5.71, *p* < .001, *d* = 0.55) were higher in the health threat manipulation than in the relaxation manipulation. The presence of the picture (P) had no influence on negative affect, disgust sensitivity, or cognitive reflection (*p*s ≥ .609) but increased positive affect compared to the no-picture (NP) condition (*M*_P_ = 24.70, *M*_NP_ = 23.03; *t*(431) = −2.21, *p* = .028, *d* = 0.21).

##### Cognitive reflection

Neither the cognitive manipulations (*M*_HT_ = 0.46, *M*_R_ = 0.42; *t*(431) = 0.74, *p* = .459, *d* = 0.07) nor the presence or absence of the picture (*M*_P_ = 0.44, *M*_NP_ = 0.44; *t*(431) = 0.16, *p* = .871, *d* = 0.02) was found to significantly affect performance on the single CRT item.

### Discussion

Experiment 2 was the second preliminary experiment. The revised manipulation check revealed large effects of the cognitive manipulations on both the personal health and the resource scarcity components of the threat score, though the effect on personal health threat perceptions was larger. The presence of the pictures was not found to strengthen (or weaken) the manipulations. The influence of the cognitive manipulations on affect measures was consistent with Experiment 1. Likewise, no effect on cognitive performance was found.

As preliminary studies, Experiments 1 and 2 had two important limitations: (1) the threat score consisted of very few items, restricting the coverage and the potential reliability of the manipulation checks; and (2) the lack of a neutral control condition made it impossible to assess whether the effect of the cognitive manipulations was due to the health threat or the relaxation manipulation or both. To address these limitations, we conducted a large-scale validation experiment that included (1) both the main manipulation check used in Experiment 2 and an additional, more comprehensive one, and (2) both the threat and relaxation manipulations used in the first two experiments and a passive control condition to measure baseline threat perceptions. In addition, we considered individual differences in the use of protective/precautionary behaviors (e.g., whether or not being vaccinated against COVID-19) as reflections of different attitudes to risk that might influence the effectiveness of the threat manipulations. Accordingly, in Experiment 3 we tested whether participants who had received a COVID vaccination revealed higher personal health risk scores than those who refused vaccination (at the time of the study a vaccination was available to all UK citizens over 18).

## Experiment 3

### Method

Experiment 3, preregistered at the OSF (https://osf.io/qyc3e), compared the COVID-19 health threat and the relaxation manipulations to a control condition.

#### Materials and procedures

Experiment 3 compared three conditions in a between-subjects design: the COVID-19 health threat manipulation, the relaxation manipulation, and the control condition. The first two manipulations were the same as in Experiment 1. The control condition was designed to measure the baseline level of threat perceptions in the sample without any manipulation (i.e., no pictures or writing task). Median response times in the writing task were 103.1 s for the relaxation manipulation and 109.2 s for the health threat manipulation. A total of 94.9% of the text boxes contained three or more words, with an average of 8.73 words each.

To ensure that the upcoming threat perception measures were meaningful for participants in the control condition, we first prompted participants in all conditions to complete these measures by considering their “current circumstances and state of mind.” Next, on two subsequent screens, participants completed the threat perception measures that were used as manipulation checks. The main threat measure was elicited first and included revised versions of the two questions used in Experiment 2 presented in random order on a scale ranging from 0 to 100: (1) “To what extent do risks to your personal health come to your mind?” and (2) “To what extent does scarcity of material resources (such as lack of goods and services) or scarcity of financial resources (such as inadequate income or savings) come to your mind?” Averaging the scores on these two items on personal health and resource scarcity threat provided the *main threat score*.

The comprehensive threat measure, which was elicited next, included 16 items on eight threat areas distinguishing not only between health and resource scarcity threat but also between personal and public threat by repeating each of the following statements twice, ending it with either “...for myself” or “...for others in society”: “Because of the COVID-19 pandemic, there is high risk of...” (1) “not finding enough affordable food or hygiene products....,” (2) “not getting enough or timely medical help when needed...,” (3) “unemployment...,” (4) “higher debt...,” (5) “being infected with COVID-19...,” (6) “becoming severely ill with COVID-19...,” (7) “being hospitalized due to COVID-19...,” (8) “dying from COVID-19...” Participants rated how much they agreed with the statements on a scale from 1 (“strongly disagree”) to 7 (“strongly agree”). The average scores on these 16 items constituted the *comprehensive threat score* (Cronbach’s *α* = .899). The same PANAS and disgust sensitivity items used in the first two experiments were elicited afterward.

A binary (“yes” or “no”) question on vaccination status was added to the survey for additional exploratory analysis: “Have you been vaccinated against the coronavirus (COVID-19) with at least one dose?” The CRT item, explored in previous experiments and in Experiment 4, was omitted from Experiment 3 by mistake. Participants were paid a flat fee of £1 for completing the study. In the debriefing, 3.4% of participants in the threat manipulation condition (20 participants) reported experiencing distress during the experiment.

#### Sample

Because we planned to test our main hypothesis (H_5_) twice, with two different manipulation checks, we used a Bonferroni correction and set *α* = 0.025. To detect at least a small effect size (*f* = 0.10) in a one-way ANOVA model (1− *β* = 0.95), we estimated the target sample size in G*Power 3.1.9.4 (Faul et al., 2009) to be 1779 participants in total. Experiment 3 was conducted on December 2, 2021. A total of 1777 participants (age: *M* = 38.9, *SD* = 13.5) were recruited (control = 617; relaxation = 578; COVID-19 health threat = 582).

#### Hypothesis

- H_5_: The main and the comprehensive threat scores will be higher in the health threat manipulation than in both the relaxation manipulation and the control condition.

### Results

#### Confirmatory tests

**Fig. 2:**
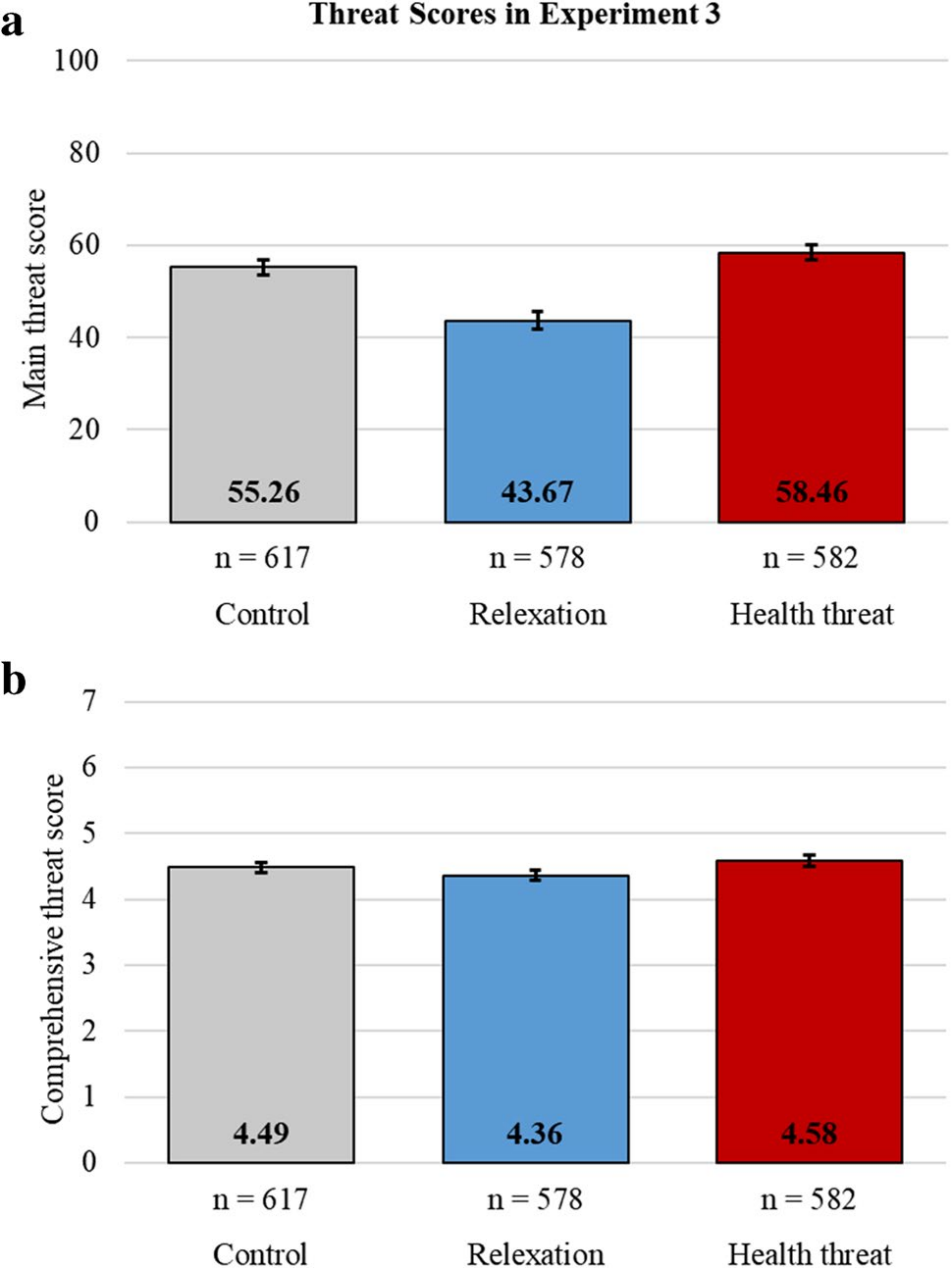
**a** Main and **b** comprehensive threat scores for the control, the relaxation manipulation, and the health threat manipulation conditions in Experiment 3. Error bars indicate 95% confidence intervals

The manipulations affected threat perceptions as predicted (H_5_) (see Fig. 2). The preregistered one-way ANOVA models on both the main (*F*(2, 1774) = 73.90, *p* < .001, η_p_^2^ = .077) and the comprehensive (*F*(2, 1774) = 7.91, *p* < .001, η_p_^2^ = .009) threat scores showed significant differences across the experimental conditions. The health threat manipulation increased the main threat scores above both the relaxation manipulation (*t*(1158) = 11.18, *p* < .001, *d* = 0.66) and the control (*t*(1197) = 2.71, *p = *.007, *d = *0.16). The comprehensive threat scores in the threat manipulation condition were significantly higher than the relaxation manipulation condition (*t*(1158) = 3.92, *p* < .001, *d* = 0.23) but not the passive control condition (*t*(1197) = 1.72, *p* = .086, *d* = 0.10). The relaxation manipulation decreased threat scores compared to the control for both the main (*t*(1193) = −8.90, *p* < .001, *d* = 0.52) and the comprehensive measures (*t*(1193) = −2.34,* p* = .020, *d* = 0.14).

#### Exploratory analysis

##### Threat perceptions

**Fig. 3:**
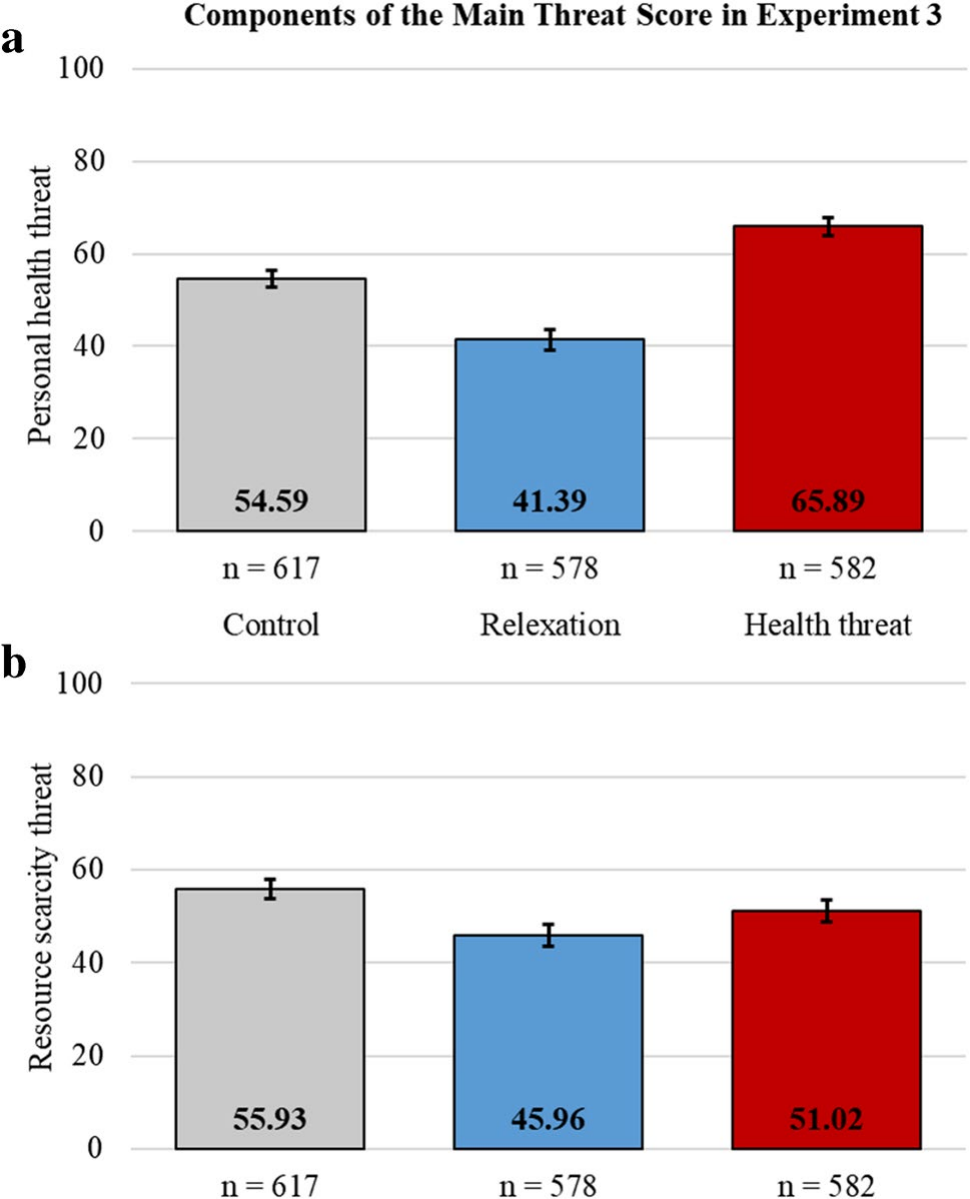
Components of the main threat perceptions score (i.e., personal health and resource scarcity threat) in the control, the relaxation manipulation, and the health threat manipulation conditions. Error bars indicate 95% confidence intervals

The cognitive manipulations had the intended effect on personal health threat but not resource scarcity threat perceptions (see Fig. 3). Compared to the control, the health threat manipulation increased and the relaxation manipulation decreased personal health threat perceptions (health threat: *t*(1197) = 7.96, *p* < .001, *d* = 0.46; relaxation: *t*(1193) = −8.90, *p* < .001, *d* = 0.51). While there was a significant difference in scarcity threat perceptions between the health threat manipulation and the relaxation manipulation (*t*(1158) = 3.00, *p* = .003, *d* = 0.18), the scarcity threat perceptions were lower than the control in both manipulation conditions (relaxation: *t*(1193) = −6.33, *p* < .001, *d* = 0.37; threat: *t*(1197) = −3.11, *p* = .002, *d* = 0.18). The components of the comprehensive threat score provided consistent results, indicating that the largest and most consistent effects of the threat manipulation were on personal health threat perceptions (see Supplementary Information).

##### Affect

The impact of the cognitive manipulations on affect was weaker but consistent with the first two experiments. In pairwise comparisons, the difference between the relaxation and health threat manipulations was significant for disgust sensitivity (*M*_HT_ = 13.45, *M*_R_ = 12.38; *t*(1158) = 2.84, *p* = .005, *d* = 0.17) and negative affect (*M*_HT_ = 17.64, *M*_*R*_ = 16.17; *t*(1158) = 3.42, *p* < .001, *d* = 0.20) but not for positive affect (*M*_HT_ = 27.36, *M*_R_ = 28.04; *t*(1158) = −1.48, *p* = .139, *d* = 0.09).

##### Vaccination

Threat perceptions can depend on vaccination status (Isler et al., 2020). Since Experiment 3 was conducted when multiple COVID-19 vaccines had been widely available in the UK for almost a year, compared to participants who reported having received at least one dose of the COVID-19 vaccine (90.9%), those who remained unvaccinated (9.1%) might have felt less threatened by the COVID-19 pandemic. Consistent with this argument, the baseline levels of perceived health threat, as measured by scores on the personal health item of the main threat measure in the control condition, were significantly lower for the unvaccinated (*M* = 46.35) than the vaccinated (*M* = 55.35), *t*(615) = −2.56, *p* = .011, *d* = 0.37. Relatedly, the effect of the cognitive manipulations on personal health threat perceptions was stronger for the vaccinated (*M*_HT_ = 67.63, *M*_*R*_ = 41.61; *t*(1049) = 16.80, *p* < .001, *d* = 1.04) than the unvaccinated participants (*M*_HT_ = 48.17, *M*_*R*_ = 39.37; *t*(107) = 1.54, *p* = .125, *d* = 0.30). Analysis of the comprehensive threat measure supports these results (see Supplementary Information).

Considering the passive control condition and vaccination status in a 3 (experimental condition) by 2 (vaccination status) two-way ANOVA models, there was no significant difference in affect among the three experimental conditions overall (*p*s ≥ .530) and no interaction with vaccination status (*p*s ≥ .112). There was no main effect of vaccination status on either positive affect (*F*(1, 1771) = 0.05, *p* = .818, η_p_^2^ < .001) or negative affect (*F*(1, 1771) = 3.79, *p* = .052, η_p_^2^ = .002), but disgust sensitivity was higher among the non-vaccinated participants (*F*(1, 1771) = 11.56, *p* < .001,* η*_*p*_^2^ = .006).

### Discussion

Confirmatory tests showed that the manipulations affected perceptions of COVID-19 threat as intended: compared to the control, the health threat manipulation increased and the relaxation manipulation decreased threat perceptions. The manipulations had a stronger and more consistent effect on personal health threat perceptions than on scarcity threat perceptions. Exploratory analysis revealed small (*d* < 0.20) differences in negative affect and disgust sensitivity but no differences in positive affect between the health threat and the relaxation manipulations.

Despite the insights of Experiment 3, it remains unknown whether the effect on personal health threat perceptions is replicable and effective beyond the COVID-19 context. Also, an effective resource scarcity threat perceptions manipulation remains lacking. Therefore, we decided to run a fourth experiment to replicate the effect of the health threat manipulation on personal health threat perceptions and to test a novel manipulation for activating personal resource scarcity threat perceptions both in the context of the COVID-19 pandemic and in general.

## Experiment 4

### Method

Experiment 4 compared both general and COVID-19-specific versions of the health threat and the resource scarcity threat manipulations to the relaxation manipulation and a passive control condition. The experiment was preregistered at the OSF (https://osf.io/h24vf).

#### Materials and procedures

Experiment 4 included six conditions in a between-subjects design: (1) the general and (2) the COVID-19-specific personal health threat manipulations, (3) the general and (4) the COVID-19-specific personal resource scarcity threat manipulation, (5) the relaxation manipulation, and (6) the control condition.

The COVID-19 health threat and the relaxation manipulations were the same as in the previous experiments using visuals (see Fig. 1a and b), except that the pronoun “you” was added to the instructions to focus attention on personal risks (e.g., “you becoming very unwell” or “you lying on your bed”). The general health threat manipulation was the same as the COVID-19 health threat manipulation except that the prompt ended with the phrase “due to a new and very serious infectious disease and needing emergency help” rather than the phrase “from COVID-19 and needing emergency help.” To activate perceptions of resource scarcity, a picture of empty shelves in a supermarket was displayed (see Fig. 1c) together with the prompt “look at the picture and think about you urgently needing essential and emergency goods but there being none available…” For the general scarcity condition, the sentence ended with “due to a new and very serious economic crisis,” whereas for the COVID-19 scarcity condition it ended with “due to COVID-19 related shortages.” The same writing tasks as in the previous experiments were implemented to complement the manipulations (see “Sample”). Median response times in the writing task were 104.5 s for the relaxation manipulation, 111.7 s for the general and 111.1 s for the COVID-19 health threat manipulations, and 115.3 s for the general and 120.9 s for the COVID-19 scarcity threat manipulations. As in Experiment 3, the control condition measured baseline rates of threat perceptions without any experimental manipulation. As in all other experiments, most text boxes (94.4%) contained three or more words, with an average of 9.72 words per text box.

Next, as in Experiment 3, all participants were prompted to answer the following questions based on their current circumstances and state of mind and were given the manipulation checks in the following two screens. The first screen included the same two questions as in the main threat measure used in Experiment 3, one on personal health threat and another on resource scarcity threat perceptions. Since our modified manipulations were specifically designed to activate personal threat perceptions, the second screen included the eight personal threat perception items from the comprehensive threat measure in Experiment 3 and excluded the remaining eight items on societal threat perception (see Section "Sample").

Using these ten items, we preregistered three main dependent variables: (1) *the personal health threat score* (Cronbach’s *α* = .861), calculated as the average of the five items about personal health threat (i.e., one item from the first screen and four items from the second screen) converted to the percent of maximum possible score (POMP; Cohen et al., 1999), (2) *the personal scarcity threat score* (Cronbach’s *α* = .782), calculated as the average of five items about personal resource scarcity threat (i.e., one item from the first screen and four items from the second screen) converted to POMP, and (3) *the personal threat score* (Cronbach’s *α* = .842), calculated as the average of the first two scores.

Next, for exploratory analysis, participants completed in two counterbalanced screens (1) the same PANAS and disgust sensitivity items used in the previous experiments and (2) a three-item four-option multiple-choice version of the Cognitive Reflection Test (Sirota & Juanchich, 2018). We used a three-item version to provide a more rigorous evaluation of our previous test showing no effect of threat on cognitive performance, which was based on a single item from the test.

Finally, answers to the same survey questions as in Experiment 3 were elicited. Participants were paid a flat fee of £1 for completing the study. In the debriefing, only 2.0% of participants (53 participants) reported experiencing some distress during the experiment.

#### Sample

We estimated our sample size based on testing of H_6_ using G*Power 3.1.9.4 (Faul et al., 2009). To detect a small main effect (*f* = 0.10) of manipulations in a one-way ANOVA with six conditions, *α* = 0.05, and 1− *β* = 0.99, the required sample size was calculated to be at least 2682 participants in total. Sensitivity analysis showed that this sample size allowed for the detection of an interaction effect size of *f = *0.05 or more with 1− *β* = 0.99 in a mixed ANOVA for testing H_7_. Experiment 4 was conducted on March 3, 2022. A total of 2689 participants (age: *M* = 38.8, *SD* = 13.4) were recruited (control = 458; relaxation = 438; general health = 439; COVID-19 health = 449; general scarcity = 452; COVID-19 scarcity = 453).

#### Hypotheses

- H_6_: The personal threat scores will be higher in the health and scarcity threat manipulations than in the relaxation manipulation and the control condition.
- H_7_: The difference in personal threat scores between the health and scarcity threat manipulations will depend on the score type (i.e., personal health vs. personal scarcity threat scores).
- H_7A_: The personal health threat scores will be higher in the health threat manipulation conditions than in the relaxation manipulation, the passive control, and the scarcity threat manipulation conditions.
- H_7B_: The personal scarcity threat scores will be higher in the scarcity threat manipulation conditions than in the relaxation manipulation, the passive control, and the health threat manipulation conditions.

### Results

#### Confirmatory tests

**Fig. 4:**
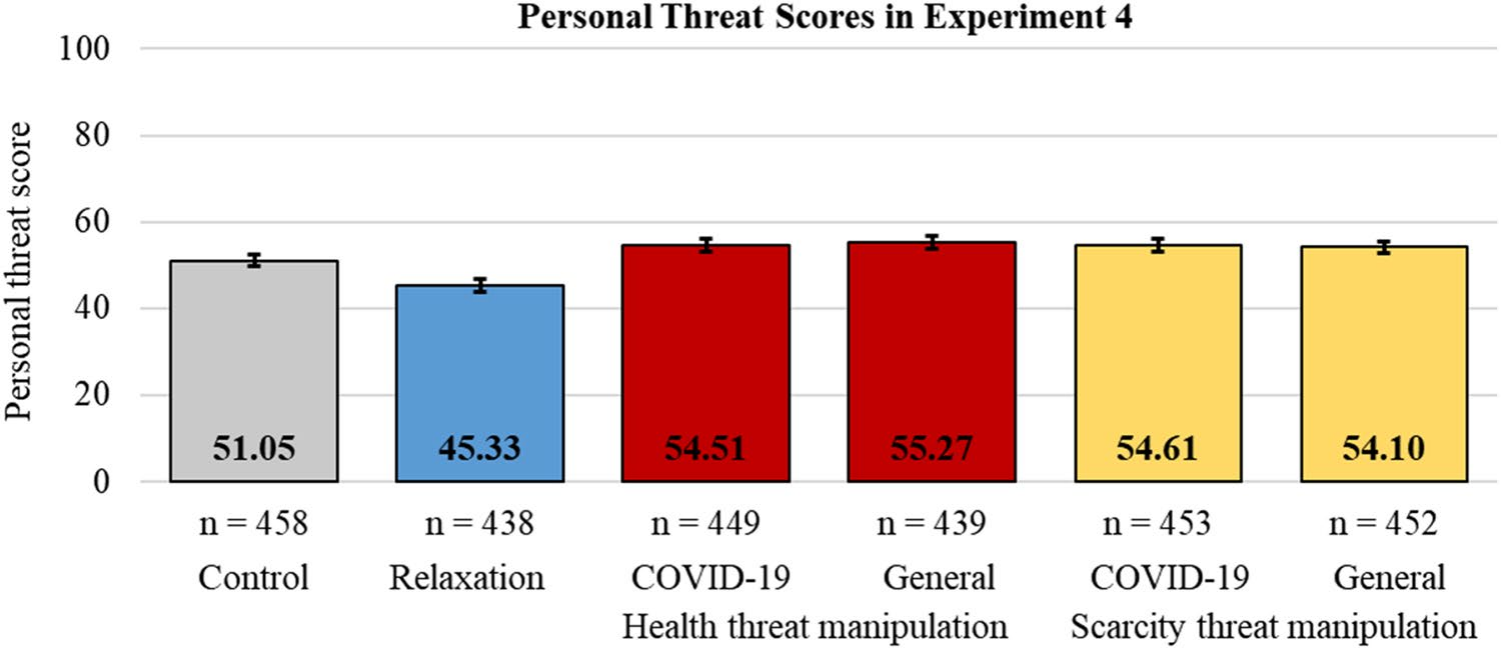
Personal threat scores for the control, the relaxation manipulation, the COVID-19-specific and the general health threat manipulation, and the COVID-19-specific and the general resource scarcity threat manipulation conditions. Error bars indicate 95% confidence intervals

The cognitive manipulations were effective (see Fig. 4), with the preregistered one-way ANOVA model showing significant differences in personal threat scores across the six conditions (*F*(5, 2683) = 26.86, *p* < .001, η_p_^2^ = .048). Supporting H_6_, all threat manipulations increased the personal threat scores above both the relaxation manipulation and the control conditions, whereas the relaxation manipulation lowered the scores below the control (see Table 1).

**Table 1:** Pairwise comparisons of (overall) personal threat scores in Experiment 4

	Control			Relaxation		
	*t*	*p*	*d*	*t*	*p*	*d*
Health threat						
COVID-19	3.50	< .001	0.23	8.90	< .001	0.60
General	4.21	< .001	0.28	9.50	< .001	0.64
Scarcity threat						
COVID-19	3.45	< .001	0.23	8.65	< .001	0.58
General	3.07	.002	0.20	8.48	< .001	0.57
Relaxation						
	−5.64	< .001	0.38			

The *t*-statistics, *p*-values, and effect sizes (Cohen’s *d*s) for preregistered two-tailed independent-samples *t*-tests comparing the personal threat scores in the cognitive manipulation conditions with the control and the relaxation manipulation conditions

**Fig. 5:**
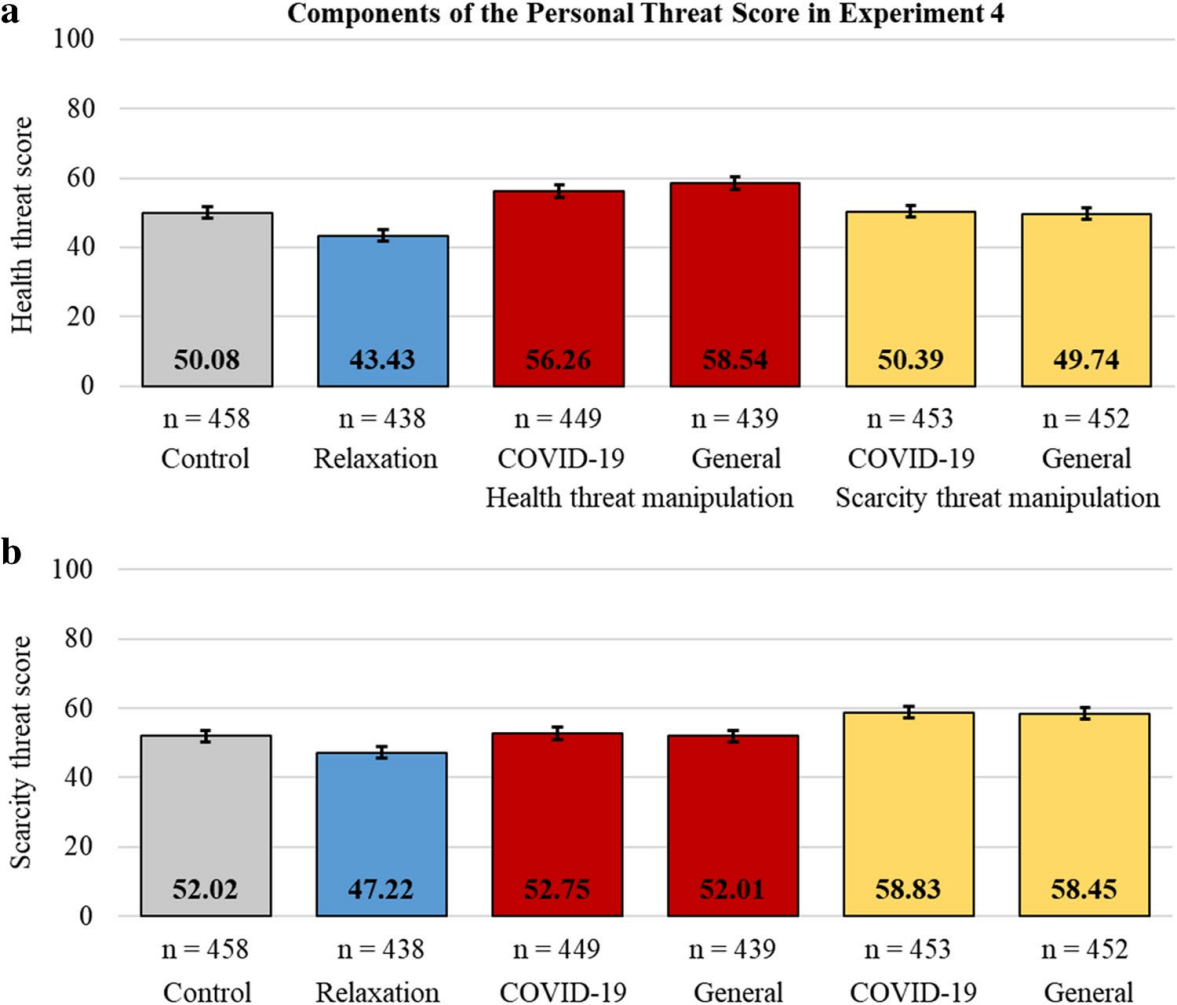
Personal health (**a**) and personal resource scarcity (**b**) threat scores for the control, the relaxation manipulation, the COVID-19-specific and the general health threat manipulation, and the COVID-19-specific and the general resource scarcity threat manipulation conditions. Error bars indicate 95% confidence intervals

Supporting H_7_ (see Fig. 5), the preregistered mixed ANOVA model indicated a significant interaction effect between the experimental conditions and the score type (*F*(5, 2683) = 44.83, *p* < .001, η_p_^2^ = .077). Specifically, (H_7A_) the personal health threat scores in the health threat manipulations were higher than all other experimental conditions and (H_7B_) the personal scarcity threat scores in the scarcity threat manipulations were higher than all other experimental conditions (see Table 2).

**Table 2:** Pairwise comparisons of personal health and resource scarcity threat scores in Experiment 4

*Health threat scores:*	Control			Relaxation			Scarcity threat					
							COVID-19			General		
	t	p	d	t	p	d	t	p	d	t	p	d
Health threat												
COVID-19	5.11	< .001	0.34	10.31	< .001	0.69	4.72	< .001	0.31	5.38	< .001	0.36
General	6.92	< .001	0.46	12.03	< .001	0.81	6.48	< .001	0.43	7.18	< .001	0.48
*Scarcity threat scores:*	Control			Relaxation			Health threat					
							COVID-19			General		
	t	p	d	t	p	d	t	p	d	t	p	d
Scarcity threat												
COVID-19	5.58	< .001	0.37	9.35	< .001	0.63	4.93	< .001	0.33	5.48	< .001	0.37
General	5.42	< .001	0.36	9.31	< .001	0.62	4.76	< .001	0.32	5.33	< .001	0.36

The table depicts the *t*-statistics, *p*-values, and effect sizes (Cohen’s *d*s) for preregistered two-tailed independent-samples *t*-tests comparing the personal health and personal resource scarcity threat scores in the threat manipulations conditions with the other experimental conditions

#### Exploratory analysis

##### Affect

As in Experiment 3, there were small differences across the experimental conditions in disgust sensitivity (*F*(5, 2683) = 7.52, *p* < .001, η_p_^2^ = .014) and negative affect (*F*(5, 2683) = 4.71, *p* < .001, η_p_^2^ = .009) but not positive affect (*F*(5, 2683) = 2.14, *p* = .058, η_p_^2^ = .004). See Table 3 for details.

**Table 3:** Affect and cognitive reflection measures in Experiment 4

	Disgust Sensitivity		Negative Affect		Positive Affect		Cognitive Reflection	
	*M*	*SD*	*M*	*SD*	*M*	*SD*	*M*	*SD*
Health threat								
COVID-19	12.82	6.62	16.59	7.62	27.54	7.64	1.45	1.19
General	13.35	6.93	17.22	7.60	27.05	8.20	1.40	1.19
Scarcity threat								
COVID-19	14.97	8.23	18.00	8.09	26.85	8.14	1.34	1.20
General	14.26	8.08	17.46	7.59	27.48	7.98	1.42	1.21
Relaxation								
	12.40	6.01	15.73	6.91	27.68	7.73	1.39	1.20
Control								
	13.67	7.37	17.06	7.58	28.42	8.25	1.39	1.19

The means (*M*) and standard deviations (*SD*) of disgust sensitivity, negative affect, positive affect, and Cognitive Reflection Test scores across the conditions in Experiment 4

##### Cognitive reflection

Consistent with the previous experiments, the cognitive manipulations had no effect on cognitive reflection, as measured by performance on the CRT (*F*(5, 2683) = 0.39, *p* = .855, *η*_*p*_^*2*^* = *.001). See Table 3 for details.

##### Vaccination

As in the previous experiment, we explored the role of vaccination status in threat perceptions. A total of 91.1% of participants in Experiment 4 reported having received at least one dose of the COVID-19 vaccine. As in Experiment 3, the personal health threat scores in the control condition were significantly lower for the unvaccinated (*M* = 43.31) than the vaccinated (*M* = 50.82), *t*(456) = −2.76, *p* = .006, *d* = 0.43. Similarly, compared to the relaxation manipulation, the combined effect of the two health threat conditions on personal health threat perceptions was stronger for the vaccinated (*M*_HT_ = 58.13, *M*_R_ = 43.39; *t*(1212) = 13.15, *p* < .001, *d* = 0.80) than the unvaccinated participants (*M*_HT_ = 49.55, *M*_R_ = 43.96; *t*(110) = 1.29, *p* = .201, *d* = 0.26). While the personal scarcity threat perceptions in the control condition were not significantly different between the unvaccinated (*M* = 53.57) and the vaccinated (*M* = 51.85; *t*(456) = 0.602, *p* = .547, *d* = 0.09), when compared to the relaxation manipulation, the combined effect of the two resource scarcity threat conditions on personal scarcity threat perceptions was stronger for the vaccinated (*M*_HT_ = 58.54, *M*_R_ = 46.36; *t*(1225) = 11.04, *p* < .001, *d* = 0.67) than the unvaccinated participants (*M*_HT_ = 59.66, *M*_R_ = 57.15; *t*(114) = 0.69, *p* = .494, *d* = 0.14).

### Discussion

Experiment 4 replicated our previous finding that the health threat manipulation specifically activates personal health threat perceptions, showed that the novel scarcity threat manipulation successfully and specifically activates personal scarcity threat perceptions, and established the validity of these manipulations both in the context of the COVID-19 pandemic and in general. Consistent with previous experiments, the influence of the cognitive manipulations on affect and cognitive reflection were either small or nonsignificant.

## Conclusion

We introduced a technique that can separately activate personal health threat or personal resource scarcity threat perceptions either in the specific context of the COVID-19 pandemic or in general. We compared these threat manipulations with a passive control and a relaxation manipulation. The former provides baseline measures of threat perceptions at the time of the study in the population under study, while the latter is useful for assessing the effects of the threat manipulation when baseline levels of perceived threat are already high in the population, as it provides a reference group with relatively weak threat perceptions. Overall, this technique provides an effective way of manipulating and assessing health and scarcity threats in experimental research, whether online or in the laboratory, thereby providing an alternative to less powerful research designs based on cross-sectional and correlational studies. The final version of the experimental materials are available as Qualtrics and PDF files in the OSF project site (https://osf.io/grafm/).

Across four experiments, we found evidence that the cognitive manipulations introduced here reliably activate threat perceptions as intended. We did not observe any issues regarding the psychological safety of the technique among our Prolific samples, as reports of psychological distress due to the threat manipulations were rare and manageable by a debriefing toolkit. However, use of the technique among the general public without any survey experience or individuals with special needs may benefit from added precautions. Finally, we note that our studies show no important differences in threat perceptions across different incentivization schemes (e.g., flat fee, bonus payments), suggesting that the technique is effective regardless of which schemes are adopted.

The effect of the threat manipulations was greater for personal than for public threat perceptions. This is in line with observed trends that preventive behavior such as vaccination is motivated more effectively with messages emphasizing personal than public benefit (Banker & Park, 2020; Milkman et al., 2021), especially when people perceive themselves to be at high risk (Isler et al., 2020). Consistent with these findings, the cognitive manipulations were found to be particularly effective among those who reported having received at least one dose of the COVID-19 vaccine. In contrast, the unvaccinated participants had lower baseline levels of threat perceptions and were less affected by the manipulations.

Exploratory analysis further suggested that the techniques create small but systematic differences in negative affect and disgust sensitivity between the threat and the relaxation manipulations, which are indicative of increased threat. In contrast, none of the four experiments showed an effect of health or scarcity threat on CRT, failing to support the idea that people use cognitive resources to suppress thoughts about these threats, leading to poorer cognitive performance (Trémolière et al., 2012, 2014; for an alternative perspective on the effect of scarcity see Isler et al., 2023). While our null results are based on larger sample sizes when compared to previous research, one should note that CRT items were elicited towards the end of the studies, after the initial effectiveness of the manipulations may have waned.

The differences observed in threat perceptions were generally consistent, but the effect sizes showed large variation, especially in the first two preliminary experiments. This variation may be due to differences in the location of the threat perception measures in the experimental protocol, with the strongest effects observed for measures that were elicited immediately after the cognitive manipulations. These findings suggest that the manipulations have an immediate strong effect that diminishes over time, particularly when there are intervening tasks between the manipulation and the dependent variable of interest. Hence, dependent variables that are of primary interest should be elicited immediately after the manipulation to maximize experimental power.

We advocate that the proper implementation of our technique requires elicitation of both the passive control and the relaxation manipulation condition together with any of the threat manipulation conditions. The elicitation of the passive control condition is important as it provides baseline measures of threat perceptions and affect at the time of data collection. In our research, the passive control conditions in Experiments 3 and 4 were insightful in determining whether the effects were driven by threat or relaxation conditions. Variation in these baseline measures can be particularly useful for longitudinal studies or experimental studies completed at different points in time. If baseline levels of perceived threat are high, then the threat manipulations may fail to induce even higher levels, such as at the height of health and resource scarcity threats posed by the COVID-19 pandemic. The elicitation of the relaxation manipulation is recommended because it allows for inducing significant differences in threat perceptions between two randomly generated groups. In short, the relaxation manipulation may be necessary in experimentally generating differences in threat perceptions, and the passive control may be necessary in interpreting the experimental results to determine whether it is the manipulation or the relaxation manipulation that is driving any reported experimental effects. Therefore, we highly recommend the implementation of both the passive control condition and the relaxation manipulation together with any of the threat manipulation techniques, rather than pairing them only with one or the other.

Although our findings are clear, this research has some limitations. First, our tests were restricted to online convenience samples. The technique can be easily implemented in the laboratory as well, but our findings are yet to be replicated in this context. We expect the controlled environment of the lab to increase the effectiveness of the manipulations. Second, although the image of the regular bed in the relaxation manipulation was originally designed in relation to the image of the hospital bed in the health threat manipulation, we use the relaxation manipulation as a common active control condition for all threat types.

The novel technique presented here can be used to activate perceptions of personal health threat or personal resource scarcity threat perceptions, both in general and in the context of the COVID-19 pandemic. Recent studies have documented correlational findings regarding the psychological and behavioral impact of health and resource scarcity threats due to the global COVID-19 pandemic. We introduce a reliable, ethical, and easy-to-use manipulation technique to activate COVID-19 and general health and scarcity threat perceptions. Future studies can use the techniques introduced in this study to experimentally test and expand these correlational findings. Additionally, they can develop novel manipulations for other psychological phenomena by employing the systematic perspective utilized here.

## Supplementary Information

Below is the link to the electronic supplementary material.

Supplementary file1 (PDF 2.28 MB)

## Data Availability

Available at the OSF project site: https://osf.io/grafm/

## Footnotes

1 We report two-tailed *t*-tests throughout the article.

## Code availability

Available at the OSF project site: https://osf.io/grafm/
