# Supplementary material for: How to activate threat perceptions in behavior research: A simple technique for inducing health and resource scarcity threats
Source: Behav Res Methods. 2024 Aug 14;56(8):8379–95. doi: 10.3758/s13428-024-02481-6 (PMC11525315; doi:10.3758/s13428-024-02481-6)
Supplement: Supplementary file 1 — Supplementary file1 (PDF 2.28 MB) [file 13428_2024_2481_MOESM1_ESM.pdf]

Online Supplementary Information for:

How to activate threat perceptions in behavior research:
A simple technique for inducing health and resource scarcity threats

Ozan Isler[1]*, Onurcan Yilmaz[2], A John Maule[3], Simon Gächter[4,5,6]*

[1] School of Economics, University of Queensland, St Lucia, Australia 4072

[2] Department of Psychology, Kadir Has University, Istanbul, Turkey 34083

[3] Leeds University Business School, University of Leeds, Leeds, UK LS2 9JT

[4] School of Economics, University of Nottingham, Nottingham, UK NG7 2RD

[5] CESifo Munich, Germany 81679

[6] IZA Bonn, Germany 53113

* Corresponding authors: o.isler@uq.edu.au; simon.gaechter@nottingham.ac.uk

Contents

Supplementary Analysis	p. 1
Experiment 1 Instructions	p. 3
Experiment 2 Instructions	p. 12
Experiment 3 Instructions	p. 22
Experiment 4 Instructions	p. 30
Debriefing toolkit	p. 40

Supplementary Analysis

Experiment 3

For exploratory analysis, we defined additional score measures based on the comprehensive threat measure items: (1) to find the *personal health threat perception score* (Cronbach's α = .879), we averaged scores on the four health-related items (i.e., statements 5 to 8) ending with "for myself"; (2) to find the *public health threat perception score* (Cronbach's α = .904), we averaged scores on the four health-related items ending with "for others in society"; (3) to find the *personal resource scarcity threat perception score* (Cronbach's α = .730), we averaged scores on the four scarcity-related items (i.e., statements 1 to 4) ending with "for myself"; and (4) to find the *public resource scarcity threat perception score* (Cronbach's α = .729), we averaged scores on the four scarcity-related items (i.e., statements 1 to 4) ending with "for others in society".

As seen in SI Fig. 1, the public threat perception scores were generally higher than the personal threat perception scores. The health threat and relaxation manipulations created significant differences in personal health threat ($t(1158) = 4.51$, $p < .001$, $d = 0.26$), public health threat ($t(1158) = 3.96$, $p < .003$, $d = 0.23$), and personal scarcity threat ($t(1158) = 3.09$, $p = .002$, $d = 0.18$) but not in public scarcity threat scores ($t(1158) = 0.05$, $p = .963$, $d < 0.01$) of the comprehensive threat measure. Compared to the control, the health threat manipulation increased ($t(1197) = 3.11$, $p = .002$, $d = 0.18$) but the relaxation manipulation did not significantly decrease ($t(1193) = -1.51$, $p = .132$, $d = 0.09$) personal health threat scores. In contrast, the relaxation manipulation decreased public health ($t(1193) = -2.52$, $p = .012$, $d = 0.15$) and personal scarcity threat scores ($t(1193) = -2.35$, $p = .019$, $d = 0.14$) compared to the control but the health threat manipulation did not significantly increase them (public health: $t(1197) = 1.50$, $p = .133$, $d = 0.09$; personal scarcity: $t(1197) = 0.87$, $p = .387$, $d = 0.05$).

Consistent with the exploratory analysis of the main threat measure, the personal health threat perceptions according to the comprehensive threat measure were significantly lower for the unvaccinated ($M = 3.46$) than the vaccinated ($M = 3.92$), $t(615) = 2.33$, $p = .020$, $d = 0.34$. Similarly, the perception manipulations affected personal health threat perceptions of the vaccinated ($M_{HT} = 4.24$, $M_R = 3.83$; $t(1049) = 4.83$, $p < .001$, $d = 0.30$) but not the unvaccinated participants ($M_{HT} = 3.14$, $M_R = 3.21$; $t(107) = -0.23$, $p = .815$, $d = 0.04$) according to the comprehensive measure.

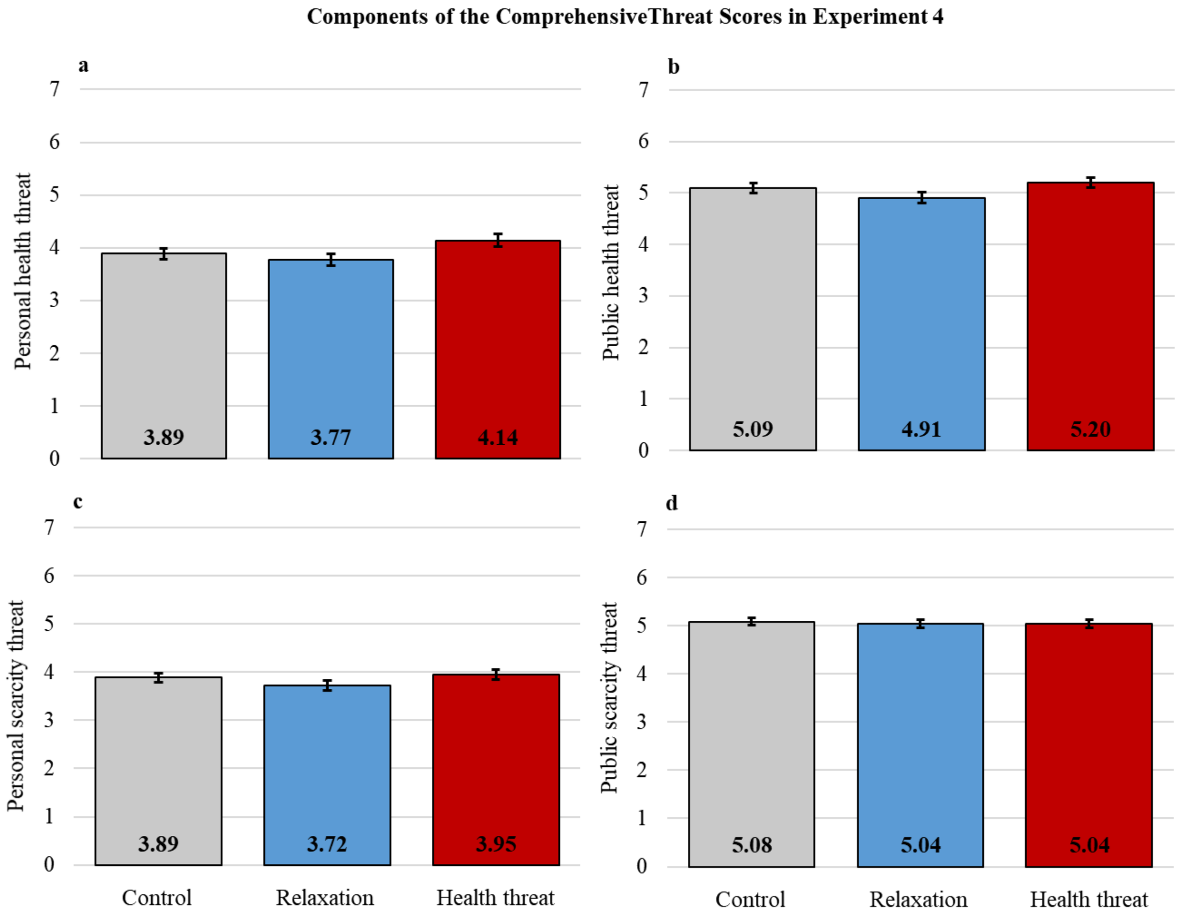

SI Fig. 1. Personal (a) and public (b) health threat and personal (c) and public (d) resource scarcity threat components of the comprehensive threat measure in the control, the relaxation manipulation, and the health threat manipulation conditions. Error bars indicate 95% confidence intervals.

Experiment 1 Instructions

Overview – No-bonus

Overview

You will complete a series of brief tasks in this study.

You will earn a participation fee of 50 pence (£0.50) for completing the study.

The next task will ask you to write four sentences about a situation.

Please continue to start the first task.

Overview – Individual bonus

Overview

You will complete a series of brief tasks in this study.

You will earn a participation fee of 50 pence (£0.50) for completing the study.

The next task will ask you to write four sentences about a situation. You will earn **50 pence in addition** to the participation fee for constructing **four full sentences**.

Please continue to start the first task.

Overview – Lottery bonus

Overview

You will complete a series of brief tasks in this study.

You will earn a participation fee of 50 pence (£0.50) for completing the study.

The next task will ask you to write four sentences about a situation. Once we have finished the study, we will randomly choose one in every ten participants. Any of these selected participants who have written **four full sentences** that are **relevant to the situation** will earn **£5 in addition** to the participation fee.

Please continue to start the first task.

COVID-19 threat manipulation

Assessment

Please look at the picture and think about **getting very unwell from COVID-19 and needing emergency medical help**.

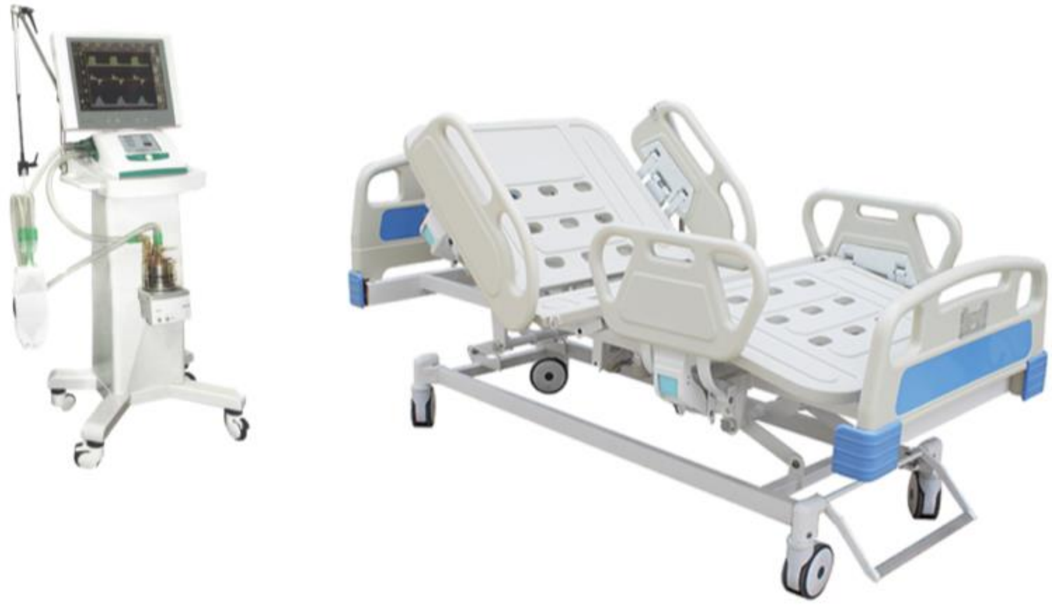

The next screen will appear in ten seconds.

[The instructions and the text boxes below appeared 10 seconds after the appearance of the picture. The picture continued to be visible.]

By typing one full sentence in each of the four boxes below, **describe what could happen to you and how you would feel in this alarming situation.**

1)

2)

3)

4)

Control condition

Assessment

Please look at the picture and think about **lying on your bed at the end of the day and feeling very relaxed**.

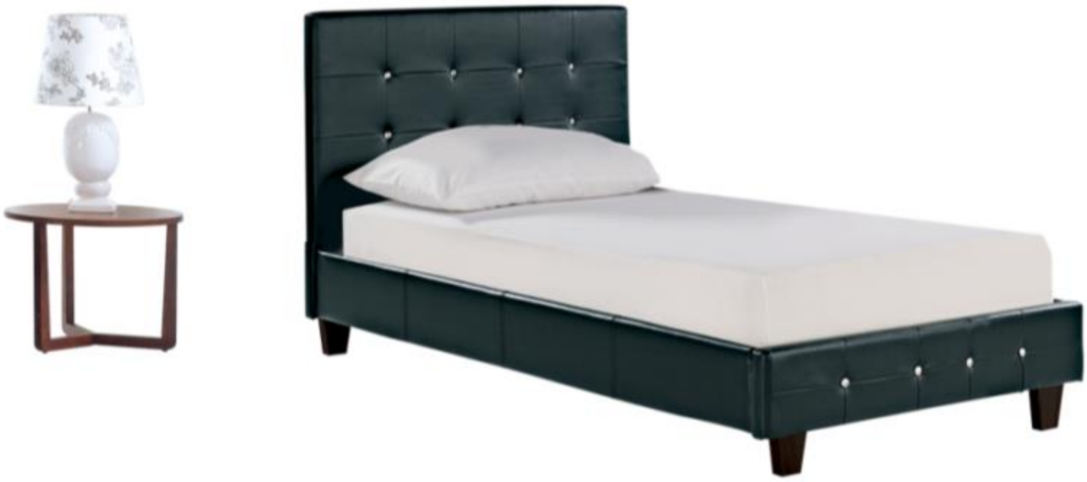

The next screen will appear in ten seconds.

[The instructions and the text boxes below appeared 10 seconds after the appearance of the picture. The picture continued to be visible.]

By typing one full sentence in each of the four boxes below, **describe what could happen to you and how you would feel in this relaxing situation.**

1)

2)

3)

4)

PANAS and disgust sensitivity

[Item order was randomized]

Below are words that describe different feelings and emotions. Please read each word and then choose the appropriate answer on the scale to indicate the extent to which you felt that way while making an assesment and completing the four sentences.

	1<br>very slightly<br>or not at all	2<br>a little	3<br>moderately	4<br>quite a bit	5<br>extremely
excited	○	○	○	○	○
scared	○	○	○	○	○
irritable	○	○	○	○	○
strong	○	○	○	○	○
attentive	○	○	○	○	○
ashamed	○	○	○	○	○

	1<br>very slightly<br>or not at all	2<br>a little	3<br>moderately	4<br>quite a bit	5<br>extremely
active	○	○	○	○	○
upset	○	○	○	○	○
guilty	○	○	○	○	○
hostile	○	○	○	○	○
proud	○	○	○	○	○
enthusiastic	○	○	○	○	○

	1<br>very slightly<br>or not at all	2<br>a little	3<br>moderately	4<br>quite a bit	5<br>extremely
alert	○	○	○	○	○
interested	○	○	○	○	○
afraid	○	○	○	○	○
disgusted	○	○	○	○	○
nervous	○	○	○	○	○
inspired	○	○	○	○	○

	1<br>very slightly<br>or not at all	2<br>a little	3<br>moderately	4<br>quite a bit	5<br>extremely
distressed	○	○	○	○	○
jittery	○	○	○	○	○
repulsed	○	○	○	○	○
determined	○	○	○	○	○

Threat perception

[Order of infection risk and severity of illness screens were randomized]

Infection risk

[Question order was randomized]

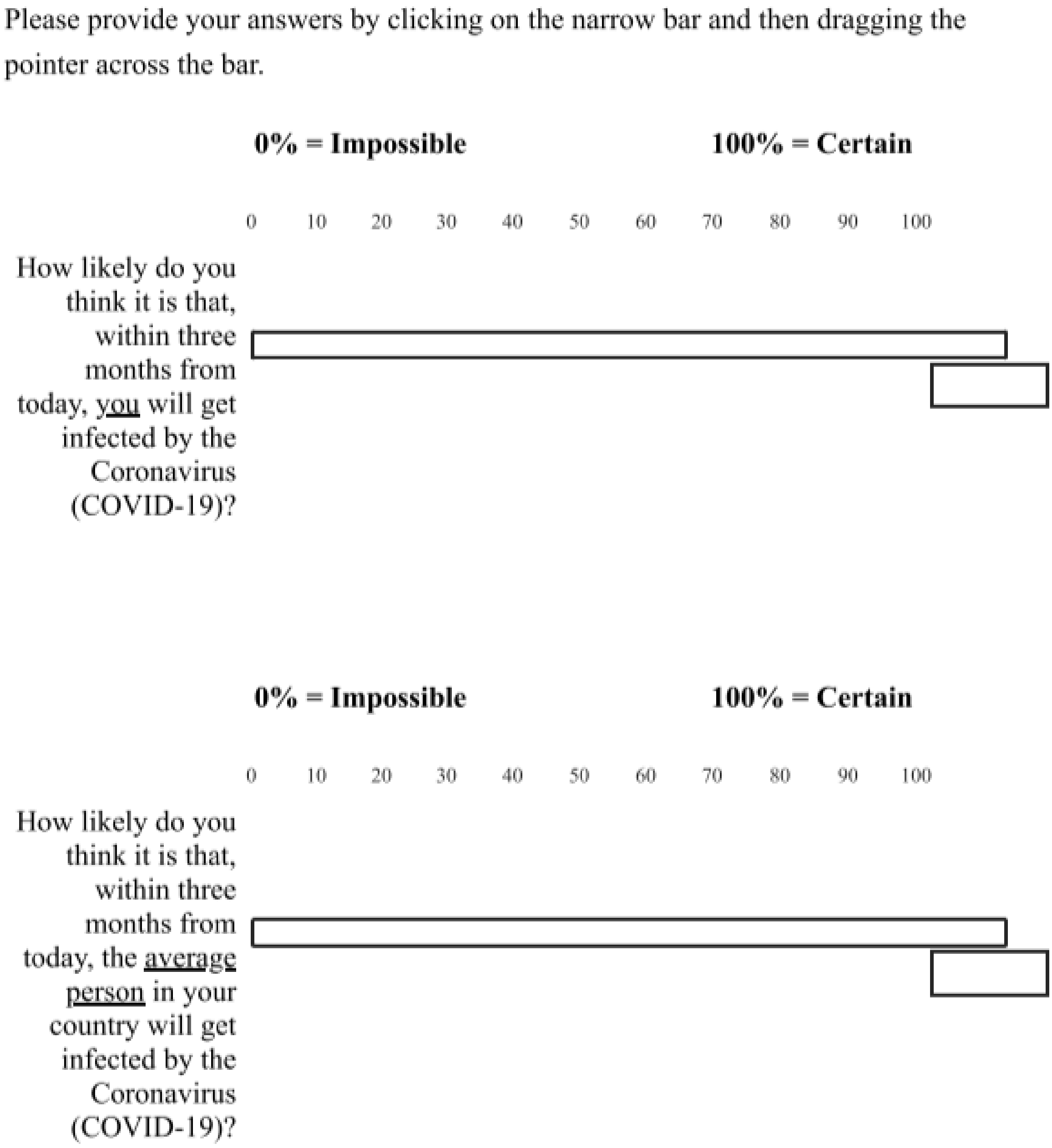

Please provide your answers by clicking on the narrow bar and then dragging the pointer across the bar.

0% = Impossible **100% = Certain**

0 10 20 30 40 50 60 70 80 90 100

How likely do you think it is that, within three months from today, <u>you</u> will get infected by the Coronavirus (COVID-19)?

0% = Impossible **100% = Certain**

0 10 20 30 40 50 60 70 80 90 100

How likely do you think it is that, within three months from today, the <u>average person</u> in your country will get infected by the Coronavirus (COVID-19)?

Severity of illness

[Question order was randomized]

Please provide your answers by clicking on the narrow bar and then dragging the pointer across the bar.

0% = Very mildly (no symptoms) **100% = Very severe (painful death)**

0 10 20 30 40 50 60 70 80 90 100

If infected by the Coronavirus (COVID-19), how severely do you think you would have the illness?

0% = Very mildly (no symptoms) **100% = Very severe (painful death)**

0 10 20 30 40 50 60 70 80 90 100

If infected by the Coronavirus (COVID-19), how severely do you think the average person in your country would have the illness?

Cognitive performance

[Answer order was randomized]

A pencil and an eraser cost £1.10 in total. The pencil costs £1.00 more than the eraser. How much does the eraser cost?

- ○ 5 pence
- ○ 1 pence
- ○ 9 pence
- ○ 10 pence

Self-reported intuition and scarcity

[Question order was randomized]

While looking at the picture and completing the four sentences...

	Not at all 0	1	2	3	4	5	6	7	8	9	Very much 10
…to what extent did you rely on your gut instinct?	○	○	○	○	○	○	○	○	○	○	○
…to what extent did scarcity of material or financial resources come to your mind?	○	○	○	○	○	○	○	○	○	○	○

Experiment 2 Instructions

Overview

Overview

Thank you for agreeing to participate in this study. You will receive 50 pence (£0.50) for completing the study, and you will have the opportunity to earn additional money.

The study consists of two different parts (Part A and Part B) involving different kinds of activities. After you complete the study, one of the parts will be randomly selected to determine your additional earnings. You will finally be asked to complete a brief survey.

You cannot participate in this study more than once.

Part A

Part A

The first task in Part A will ask you to write four sentences about a situation.

If Part A is selected for extra payments and if you have written **four full sentences** that are **relevant to the situation**, then you will earn 50 pence in addition to the participation fee.

Please continue to start the first task.

COVID-19 threat manipulation

Picture

Assessment

Please look at the picture and think about **getting very unwell from COVID-19 and needing emergency medical help**.

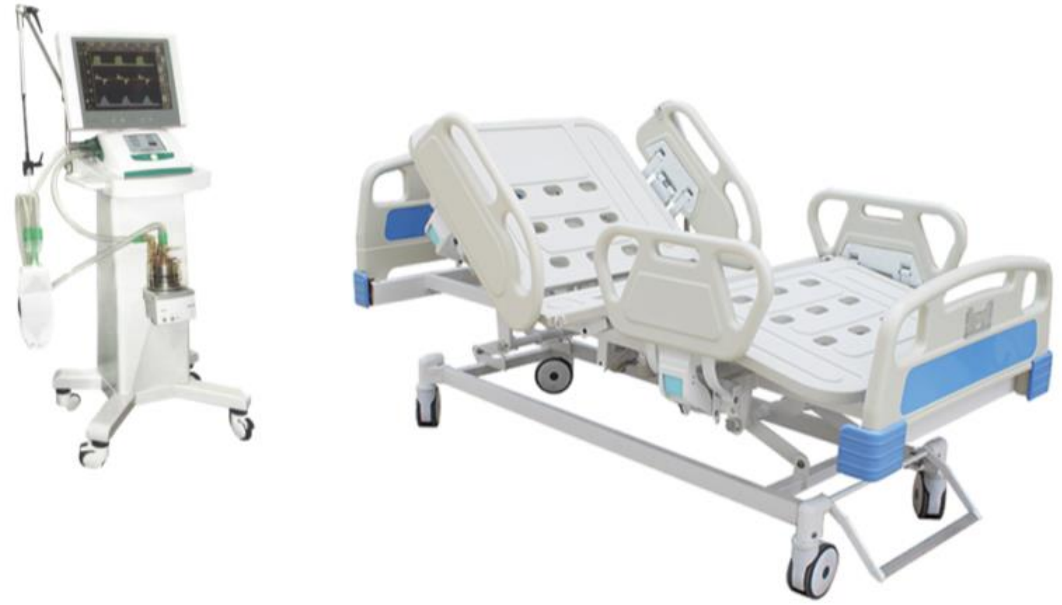

The next screen will appear in ten seconds.

[The instructions and the text boxes below appeared 10 seconds after the appearance of the picture. The picture continued to be visible.]

By typing one full sentence in each of the four boxes below, **describe what could happen to you and how you would feel in this alarming situation.**

1)

2)

3)

4)

No-picture

Please think about **getting very unwell from COVID-19 and needing emergency medical help**.

The next screen will appear in ten seconds.

[The instructions and the text boxes below appeared 10 seconds after the appearance of the instructions above. The instructions above continued to be visible.]

By typing one full sentence in each of the four boxes below, **describe what could happen to you and how you would feel in this alarming situation.**

1)

2)

3)

4)

Control condition

Picture

Assessment

Please look at the picture and think about **lying on your bed at the end of the day and feeling very relaxed**.

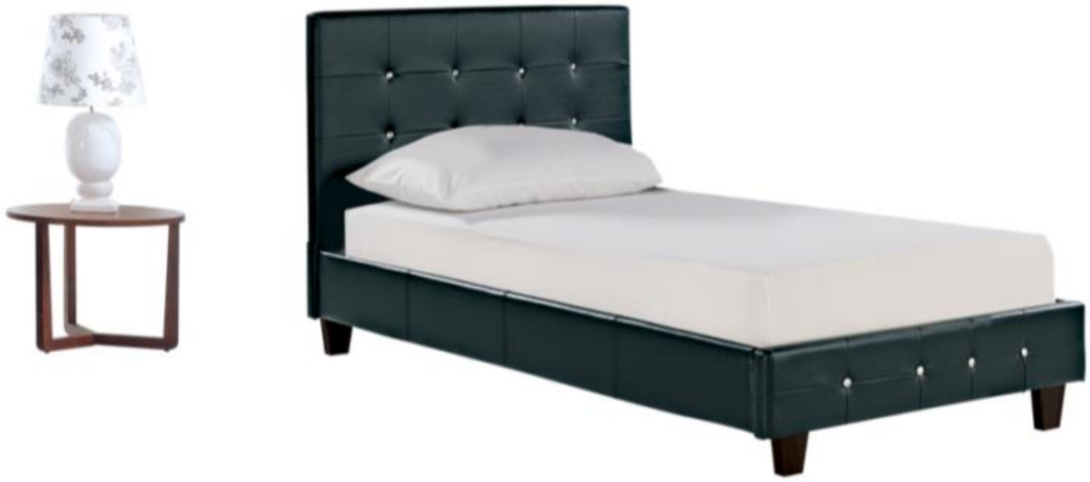

The next screen will appear in ten seconds.

[The instructions and the text boxes below appeared 10 seconds after the appearance of the picture. The picture continued to be visible.]

By typing one full sentence in each of the four boxes below, **describe what could happen to you and how you would feel in this relaxing situation.**

1)

2)

3)

4)

No-picture

Assessment

Please think about **lying on your bed at the end of the day and feeling very relaxed**.

The next screen will appear in ten seconds.

[The instructions and the text boxes below appeared 10 seconds after the appearance of the instructions above. The instructions above continued to be visible.]

By typing one full sentence in each of the four boxes below, **describe what could happen to you and how you would feel in this relaxing situation.**

1)

2)

3)

4)

Threat perception

[Question order was randomized]

Please provide your answers by clicking on the narrow bar and then dragging the pointer across the bar.

While making an assessment and trying to construct sentences...

0% = Not at all **100% = Very much**

0 10 20 30 40 50 60 70 80 90 100

0 10 20 30 40 50 60 70 80 90 100

...to what extent did risks to your personal health come to your mind?

0% = Not at all **100% = Very much**

0 10 20 30 40 50 60 70 80 90 100

...to what extent did scarcity of material resources (such as lack of goods and services) or scarcity of financial resources (such as a inadequate income or savings) come to your mind?

PANAS and disgust sensitivity

[Item order was randomized]

Below are words that describe different feelings and emotions. Please read each word and then choose the appropriate answer on the scale to indicate the extent to which you felt that way while making an assesment and completing the four sentences.

	1 very slightly or not at all	2 a little	3 moderately	4 quite a bit	5 extremely
excited	○	○	○	○	○
scared	○	○	○	○	○
irritable	○	○	○	○	○
strong	○	○	○	○	○
attentive	○	○	○	○	○
ashamed	○	○	○	○	○

	1 very slightly or not at all	2 a little	3 moderately	4 quite a bit	5 extremely
active	○	○	○	○	○
upset	○	○	○	○	○
guilty	○	○	○	○	○
hostile	○	○	○	○	○
proud	○	○	○	○	○
enthusiastic	○	○	○	○	○

	1 very slightly or not at all	2 a little	3 moderately	4 quite a bit	5 extremely
alert	○	○	○	○	○
interested	○	○	○	○	○
afraid	○	○	○	○	○
disgusted	○	○	○	○	○
nervous	○	○	○	○	○
inspired	○	○	○	○	○

	1 very slightly or not at all	2 a little	3 moderately	4 quite a bit	5 extremely
distressed	○	○	○	○	○
jittery	○	○	○	○	○
repulsed	○	○	○	○	○
determined	○	○	○	○	○

Part B

Part B

You have completed Part A.

In the only task in Part B, we will ask you a question that has a correct answer.

If Part B is selected for extra payments and if you have **correctly answered this question**, then you will earn 50 pence in addition to the participation fee.

Please continue to see the question.

Cognitive performance

[Answer order was randomized]

A pencil and an eraser cost £1.10 in total. The pencil costs £1.00 more than the eraser. How much does the eraser cost?

- ○ 5 pence
- ○ 1 pence
- ○ 9 pence
- ○ 10 pence

Experiment 3 Instructions

Overview

Overview

Thank you for agreeing to participate in this study. You will receive £1 for completing the study.

You cannot participate in this study more than once.

Passive control condition

[There was no picture or writing task in the passive control condition and the next screen appeared after 1 second.]

Active control condition

Assessment

Please look at the picture and think about **lying on your bed at the end of the day and feeling very relaxed**.

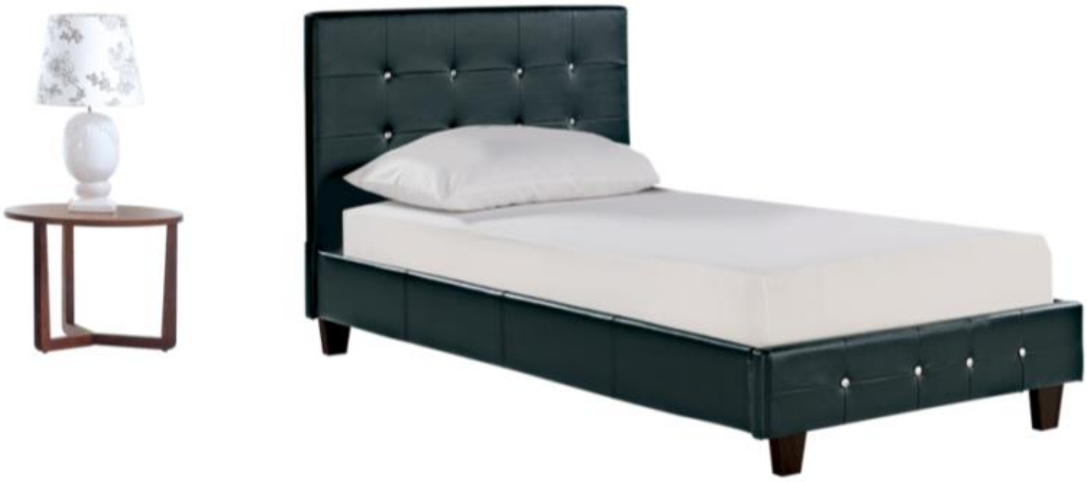

The next screen will appear in ten seconds.

[The instructions and the text boxes below appeared 10 seconds after the appearance of the picture. The picture continued to be visible.]

By typing one full sentence in each of the four boxes below, **describe what could happen to you and how you would feel in this relaxing situation.**

1)

2)

3)

4)

COVID-19 threat manipulation

Assessment

Please look at the picture and think about **getting very unwell from COVID-19 and needing emergency medical help**.

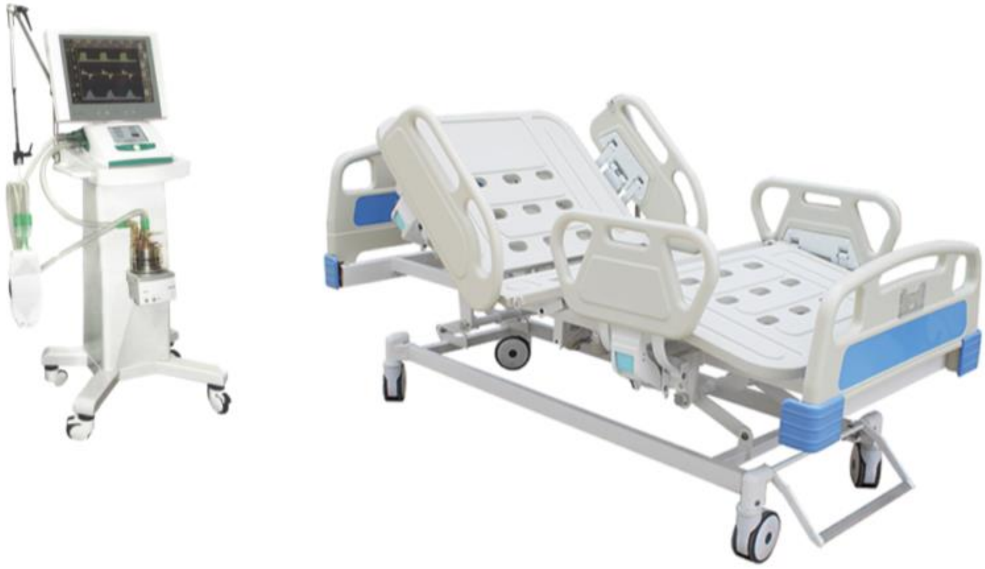

The next screen will appear in ten seconds.

[The instructions and the text boxes below appeared 10 seconds after the appearance of the picture. The picture continued to be visible.]

By typing one full sentence in each of the four boxes below, **describe what could happen to you and how you would feel in this alarming situation.**

1)

2)

3)

4)

Interim instructions

[The following instructions were displayed after all conditions for the manipulation check questions to make sense for those participants in the passive control condition who did not receive the manipulation.]

Please answer the questions on the following screens by considering your current circumstances and state of mind.

Basic threat perception

[Question order was randomized]

Please provide your answers by clicking on the narrow bar and then dragging the pointer across the bar.

0% = Not at all **100% = Very much**

0 10 20 30 40 50 60 70 80 90 100

0 10 20 30 40 50 60 70 80 90 100

...to what extent did risks to your personal health come to your mind?

0% = Not at all **100% = Very much**

0 10 20 30 40 50 60 70 80 90 100

...to what extent did scarcity of material resources (such as lack of goods and services) or scarcity of financial resources (such as a inadequate income or savings) come to your mind?

Comprehensive threat perception

[Block order was randomized]

Please indicate how much you agree with the following statements.

Because of the COVID-19 pandemic, there is high risk of...

…not finding enough affordable food or hygiene products...

	1 Strongly disagree	2 Disagree	3 Somewhat disagree	4 Neither agree nor disagree	5 Somewhat agree	6 Agree	7 Strongly agree
…for myself.	O	O	O	O	O	O	O
…for others in society.	O	O	O	O	O	O	O

…not getting enough or timely medical help when needed...

	1 Strongly disagree	2 Disagree	3 Somewhat disagree	4 Neither agree nor disagree	5 Somewhat agree	6 Agree	7 Strongly agree
…for myself.	O	O	O	O	O	O	O
…for others in society.	O	O	O	O	O	O	O

…unemployment...

	1 Strongly disagree	2 Disagree	3 Somewhat disagree	4 Neither agree nor disagree	5 Somewhat agree	6 Agree	7 Strongly agree
…for myself.	O	O	O	O	O	O	O

	1 Strongly disagree	2 Disagree	3 Somewhat disagree	4 Neither agree nor disagree	5 Somewhat agree	6 Agree	7 Strongly agree
…for others in society.	○	○	○	○	○	○	○

...higher debt...

	1 Strongly disagree	2 Disagree	3 Somewhat disagree	4 Neither agree nor disagree	5 Somewhat agree	6 Agree	7 Strongly agree
…for myself.	○	○	○	○	○	○	○
…for others in society.	○	○	○	○	○	○	○

...being infected with COVID-19...

	1 Strongly disagree	2 Disagree	3 Somewhat disagree	4 Neither agree nor disagree	5 Somewhat agree	6 Agree	7 Strongly agree
…for myself.	○	○	○	○	○	○	○
…for others in society.	○	○	○	○	○	○	○

...becoming severely ill with COVID-19...

	1 Strongly disagree	2 Disagree	3 Somewhat disagree	4 Neither agree nor disagree	5 Somewhat agree	6 Agree	7 Strongly agree
…for myself.	○	○	○	○	○	○	○
…for others in society.	○	○	○	○	○	○	○

...being hospitalised due to COVID-19...

	1 Strongly disagree	2 Disagree	3 Somewhat disagree	4 Neither agree nor disagree	5 Somewhat agree	6 Agree	7 Strongly agree
…for myself.	○	○	○	○	○	○	○
…for others in society.	○	○	○	○	○	○	○

...dying from COVID-19...

	1 Strongly disagree	2 Disagree	3 Somewhat disagree	4 Neither agree nor disagree	5 Somewhat agree	6 Agree	7 Strongly agree
…for myself.	○	○	○	○	○	○	○
…for others in society.	○	○	○	○	○	○	○

PANAS and disgust sensitivity

[Item order was randomized]

Below are words that describe different feelings and emotions. Please read each word and then choose the appropriate answer on the scale to indicate the extent to which you currently feel that way.

	1 very slightly or not at all	2 a little	3 moderately	4 quite a bit	5 extremely
alert	○	○	○	○	○
irritable	○	○	○	○	○
proud	○	○	○	○	○
ashamed	○	○	○	○	○
guilty	○	○	○	○	○
disgusted	○	○	○	○	○

	1 very slightly or not at all	2 a little	3 moderately	4 quite a bit	5 extremely
interested	○	○	○	○	○
nervous	○	○	○	○	○
inspired	○	○	○	○	○
attentive	○	○	○	○	○
afraid	○	○	○	○	○
scared	○	○	○	○	○

	1 very slightly or not at all	2 a little	3 moderately	4 quite a bit	5 extremely
excited	○	○	○	○	○
determined	○	○	○	○	○
active	○	○	○	○	○
strong	○	○	○	○	○
repulsed	○	○	○	○	○
hostile	○	○	○	○	○

	1 very slightly or not at all	2 a little	3 moderately	4 quite a bit	5 extremely
jittery	○	○	○	○	○
distressed	○	○	○	○	○
enthusiastic	○	○	○	○	○
upset	○	○	○	○	○

Experiment 4 Instructions

Overview

Overview

Thank you for agreeing to participate in this study. You will receive £1 for completing the study.

You cannot participate in this study more than once.

Passive control condition

[There was no picture or writing task in the passive control condition and the next screen appeared after 1 second.]

Active control condition

Assessment

Please look at the picture and think about **lying on your bed at the end of the day and feeling very relaxed**.

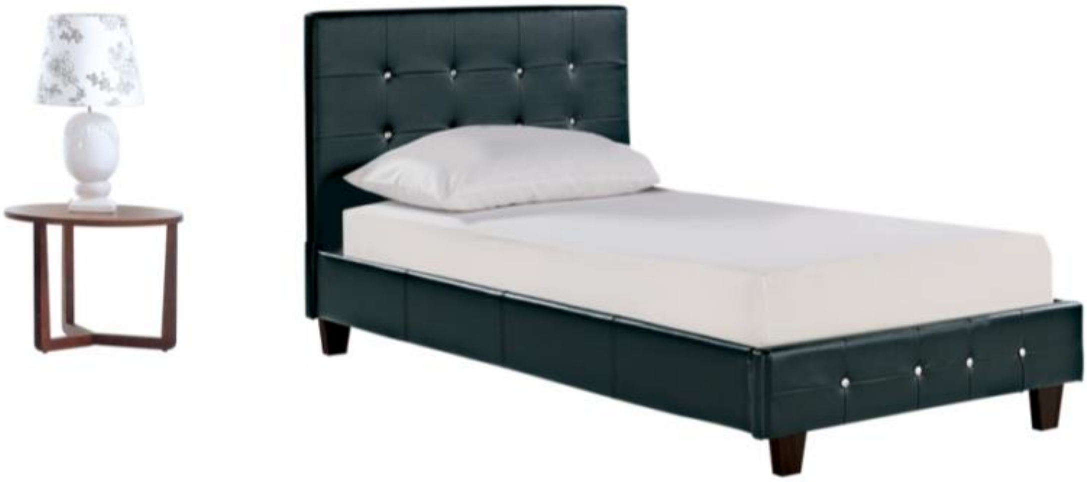

The next screen will appear in ten seconds.

[The instructions and the text boxes below appeared 10 seconds after the appearance of the picture. The picture continued to be visible.]

By typing one full sentence in each of the four boxes below, **describe what could happen to you and how you would feel in this relaxing situation.**

1)

2)

3)

4)

COVID-19 health threat manipulation

Assessment

Please look at the picture and think about **you getting very unwell from COVID-19 and needing emergency medical help**.

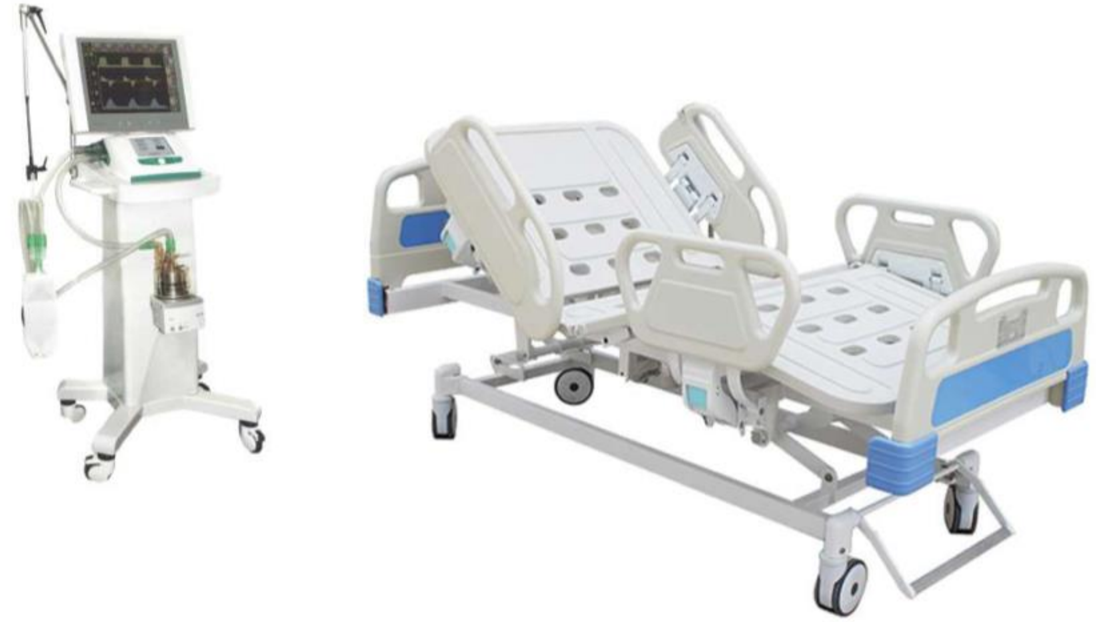

The next screen will appear in ten seconds.

[The instructions and the text boxes below appeared 10 seconds after the appearance of the picture. The picture continued to be visible.]

By typing one full sentence in each of the four boxes below, **describe what could happen to you and how you would feel in this alarming situation.**

1)

2)

3)

4)

Generic health threat manipulation

Assessment

Please look at the picture and think about **you getting very unwell due a new and very serious infectious disease and needing emergency medical help**.

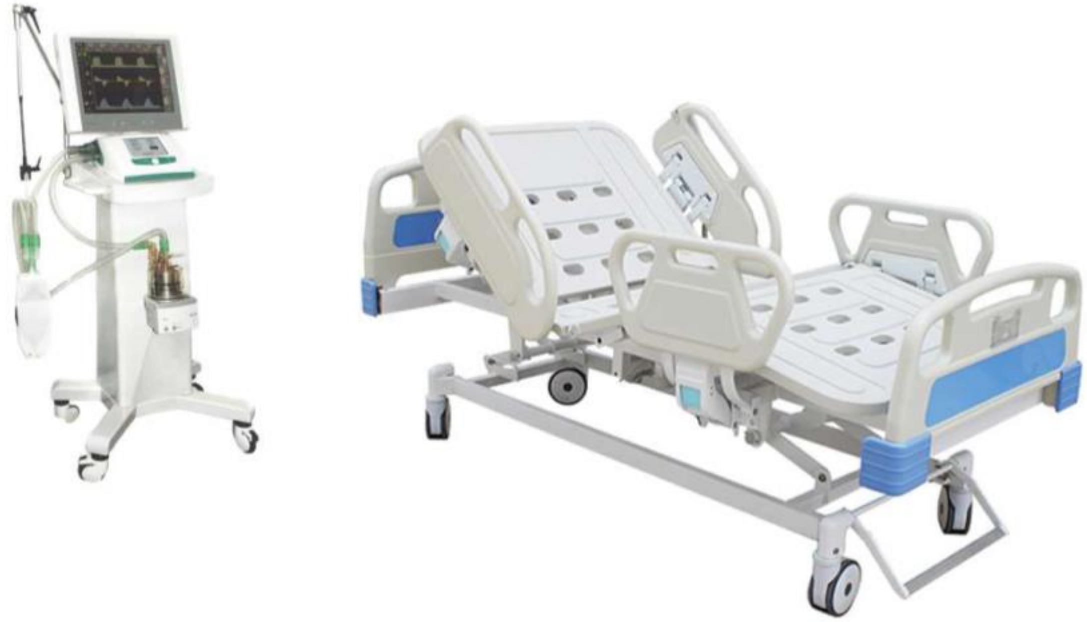

The next screen will appear in ten seconds.

[The instructions and the text boxes below appeared 10 seconds after the appearance of the picture. The picture continued to be visible.]

By typing one full sentence in each of the four boxes below, **describe what could happen to you and how you would feel in this alarming situation.**

1)

2)

3)

4)

COVID-19 scarcity threat manipulation

Assessment

Please look at the picture and think about **you urgently needing essential and emergency goods but there being none available due to COVID-19 related shortages**.

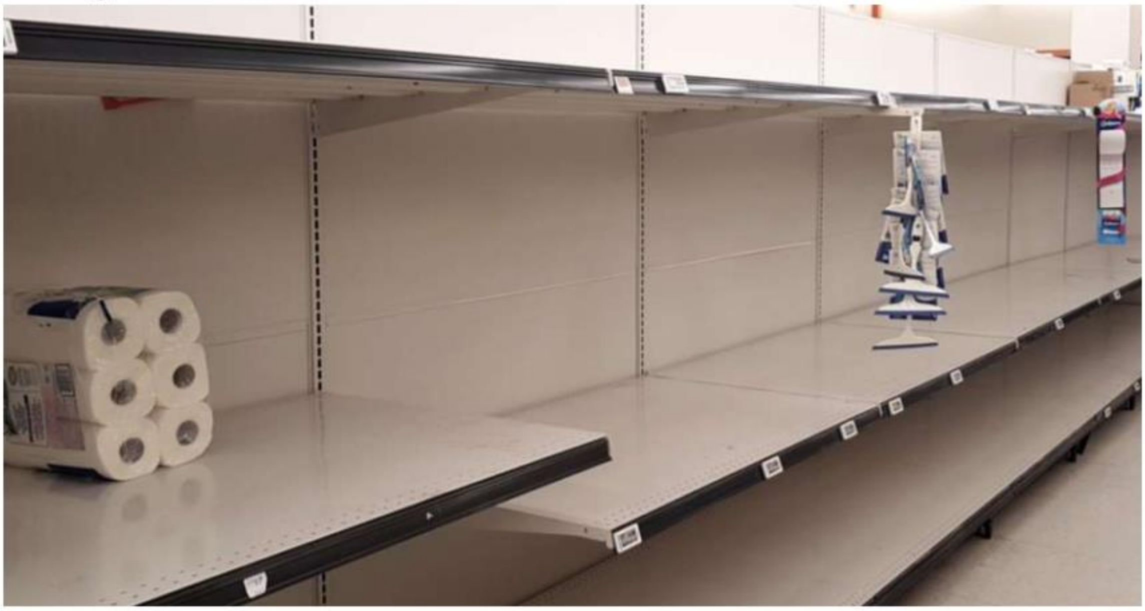

The next screen will appear in ten seconds.

[The instructions and the text boxes below appeared 10 seconds after the appearance of the picture. The picture continued to be visible.]

By typing one full sentence in each of the four boxes below, **describe what could happen to you and how you would feel in this alarming situation.**

1)

2)

3)

4)

Generic scarcity threat manipulation

Assessment

Please look at the picture and think about **you urgently needing essential and emergency goods but there being none available due to a new and very serious economic crisis**.

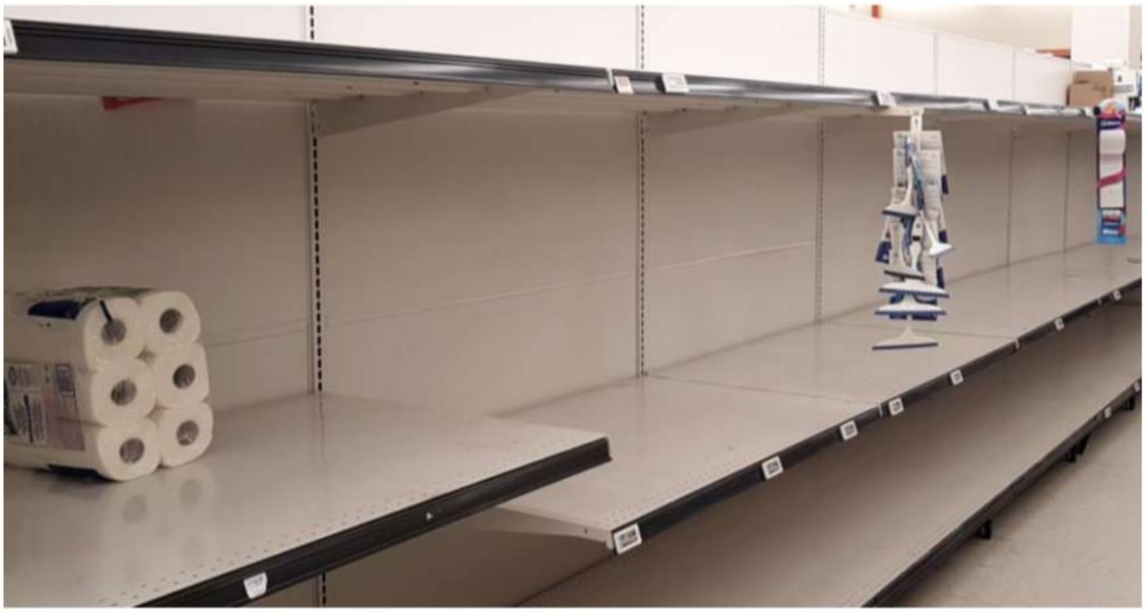

The next screen will appear in ten seconds.

[The instructions and the text boxes below appeared 10 seconds after the appearance of the picture. The picture continued to be visible.]

By typing one full sentence in each of the four boxes below, **describe what could happen to you and how you would feel in this alarming situation.**

1)

2)

3)

4)

Interim instructions

[The following instructions were displayed after all conditions for the manipulation check questions to make sense for those participants in the passive control condition who did not receive the manipulation.]

Please answer the questions on the following screens by considering your current circumstances and state of mind.

Basic threat perception

[Question order was randomized]

Please provide your answers by clicking on the narrow bar and then dragging the pointer across the bar.

0% = Not at all **100% = Very much**

0 10 20 30 40 50 60 70 80 90 100

0 10 20 30 40 50 60 70 80 90 100

...to what extent did risks to your personal health come to your mind?

0% = Not at all **100% = Very much**

0 10 20 30 40 50 60 70 80 90 100

...to what extent did scarcity of material resources (such as lack of goods and services) or scarcity of financial resources (such as a inadequate income or savings) come to your mind?

Comprehensive personal threat perception

[Block order was randomized]

Please indicate how much you agree with the following statements.

There is high risk for myself of...

	1 Strongly disagree	2 Disagree	3 Somewhat disagree	4 Neither agree nor disagree	5 Somewhat agree	6 Agree	7 Strongly agree
...dying from an infectious disease.	○	○	○	○	○	○	○
...becoming severely ill.	○	○	○	○	○	○	○
...being hospitalised.	○	○	○	○	○	○	○
...being infected with a virus.	○	○	○	○	○	○	○

There is high risk for myself of...

	1 Strongly disagree	2 Disagree	3 Somewhat disagree	4 Neither agree nor disagree	5 Somewhat agree	6 Agree	7 Strongly agree
…unemployment.	○	○	○	○	○	○	○
…not getting enough or timely medical help when needed.	○	○	○	○	○	○	○
…not finding enough affordable food or hygiene products.	○	○	○	○	○	○	○
...higher debt.	○	○	○	○	○	○	○

CRT

Please try to correctly answer the next three questions.

A bat and a ball cost £1.10 in total. The bat costs £1.00 more than the ball. How much does the ball cost?

- ○ 1 pence
- ○ 5 pence
- ○ 10 pence
- ○ 9 pence

If it takes 5 machines 5 minutes to make 5 widgets, how long would it take 100 machines to make 100 widgets?

- ○ 100 minutes
- ○ 500 minutes
- ○ 5 minutes
- ○ 20 minutes

In a lake, there is a patch of lily pads. Every day, the patch doubles in size. If it takes 48 days for the patch to cover the entire lake, how long would it take for the patch to cover half of the lake?

- ○ 24 days
- ○ 12 days
- ○ 36 days
- ○ 47 days

PANAS and disgust sensitivity

[Item order was randomized]

Below are words that describe different feelings and emotions. Please read each word and then choose the appropriate answer on the scale to indicate the extent to which you currently feel that way.

	1 very slightly or not at all	2 a little	3 moderately	4 quite a bit	5 extremely
alert					
irritable					
proud					
ashamed					
guilty					
disgusted					

	1 very slightly or not at all	2 a little	3 moderately	4 quite a bit	5 extremely
interested					
nervous					
inspired					
attentive					
afraid					
scared					

	1 very slightly or not at all	2 a little	3 moderately	4 quite a bit	5 extremely
excited					
determined					
active					
strong					
repulsed					
hostile					

	1 very slightly or not at all	2 a little	3 moderately	4 quite a bit	5 extremely
jittery					
distressed					
enthusiastic					
upset					

Debriefing Toolkit

[The following instructions were used to debrief participants and to provide help for any participant who experienced psychologic distress due to the threat manipulations.]

Thank you for participating in our study.

For scientific research purposes, you may have been asked to think about contracting COVID-19 in this study. Did this or any other aspect of this study significantly upset or distressed you? If so, please describe.

The purpose of this study is to examine the effect of thinking about the coronavirus (COVID-19) on decision making.

If you have any questions or concerns or would like a summary of the results when they are published then please contact Dr Onurcan Yilmaz (onurcan.yilmaz@khas.edu.tr).

Because some conditions in this study involved thinking about the coronavirus (COVID-19) outbreak and how it has impacted on your life, there is a small chance that you may have become distressed or upset. In that case please read the Wellness Sheet below. If you feel you need to seek support you are encouraged to contact Samaritans (telephone 116 123) or CALM (telephone 0800 58 58 58 or online support www.thecalmzone.net/help/webchat/). Otherwise, please click "Finish" to end the study.

Wellness Sheet

Show the Wellness Sheet ○ Hide the Wellness Sheet ○

Wellness Sheet

If you are unable to contact anyone (for example, if you have no access to a phone or if it is taking too long for someone to answer), it may be helpful to try to reduce your distress by taking some of the following steps:

a) Our moods often reflect the environment we are in, so, if necessary, move to an environment that is calming, warm, and if possible, uplifting.
b) Stay warm. Rug up if you have to.
c) Try some deep breathing. Put your hand on your stomach. Take a deep breath and slow down. Breathe in (to a count of 4), hold your breath (for 2 counts) and then breathe out (for a count of 6). That is, breathe out for longer than you breathe in. Deep breathing should be the lungs all the way down, so make sure your hand is rising and falling as you breathe. Shallow breathing doesn't help much and can even make you more anxious. Deep breathe slowly and consciously, following this pattern. Concentrate on your counting. Focus your mind on this for 3-5 minutes. Allow your feelings to settle.
d) Once calmer, do something you would normally soothing, such as having a cup of tea or reading the paper.
e) Decide the best way to contact the most appropriate person listed in the contacts above or a friend or family member.
f) It is important that you talk to someone, even if your distress subsides and you start to feel OK. If you are feeling OK, this may not need to be immediately, but you should try to talk to someone today.

If this information might be of use to you then you might want to copy and paste it into a document before closing your browser window.
